# Personalized mapping of inhibitory spinal cord circuits in humans via noninvasive neural decoding and in silico modeling

**DOI:** 10.1126/sciadv.adz5524

**Published:** 2025-09-19

**Authors:** Alejandro Pascual-Valdunciel, Natalia T. Cónsul, Robert M. Brownstone, Marco Beato, Dario Farina, Filipe Nascimento, M. Görkem Özyurt

**Affiliations:** ^1^ Department of Bioengineering, Imperial College London, London, UK.; ^2^ BSICoS group, I3A Institute, University of Zaragoza, IIS, Aragon, Zaragoza, Spain.; ^3^ Department of Neuromuscular Diseases, UCL Queen Square Institute of Neurology, University College London, London WC1N 3BG, UK.; ^4^ Department of Neuroscience Physiology and Pharmacology (NPP), Gower Street, University College London, London WC1E 6BT, UK.

## Abstract

Studying human motoneuron activity through electromyography (EMG) can yield insights into the operation of fundamental spinal cord microcircuits. Traditional surface and needle EMG methodologies have limited capacity to shed light on the diversity of motor unit (MU) control strategies that may be unique to each individual. Here, we used high-density surface EMG (HDsEMG) to sample multiple MUs per participant to investigate the features of inhibitory spinal microcircuits in both upper and lower limb control. We characterized the net inhibition as a function of individual MU firing rates, revealing participant-specific relationships. In silico modeling replicated these experimental characteristics and suggested that properties of the inhibitory currents rather than motoneuron size are responsible for net functional inhibition. Our results show that HDsEMG can highlight distinct control strategies across circuits and motor pools, revealing participant-specific properties of inhibitory spinal microcircuits.

## Teaser

High-density surface EMG electrodes can reveal the functional properties of inhibitory spinal circuits.

## INTRODUCTION

Movement relies on the precise coordination of muscle contractions, enabling fundamental actions like grasping and walking. Muscles are composed of individual fibers innervated by spinal motoneurons. A single motoneuron connects to a group of muscle fibers, collectively forming a motor unit (MU). While large parts of the central nervous system (CNS) are responsible for various aspects of movement, motoneurons serve as the “final common pathway” responsible for its execution (*1*).

Electromyography (EMG) is a valuable tool for studying motoneurons and their associated circuits. EMG can be used to record gross muscle activity, reflecting neuronal activity in the population (pool) of motoneurons innervating that muscle through global surface EMG (sEMG) or intramuscular EMG (iEMG). In addition, the activity of individual MUs can be recorded via single unit iEMG, which reflects the action potentials of individual motoneurons. Hence, motoneurons are the most readily recordable cell type in the human CNS, offering a valuable window into CNS activity.

The region of the CNS that is responsible for organizing the pattern of muscle contractions for limb movement is the spinal cord. Various fundamental spinal circuits have been identified in animal studies, with some also characterized in humans. One well-characterized example is the circuit controlling reciprocal inhibition between antagonist muscles, mediated by Ia inhibitory interneurons (*2*, *3*). This circuit, also responsible for inhibition of antagonist muscles in stretch reflexes, plays a key role in ensuring proper flexor-extensor alternation during locomotion (*4*). Another example is the circuit responsible for producing a “cutaneous silent period” (CSP) (*5*), a transient pause in voluntary muscle contraction produced by electrical stimulation of a cutaneous nerve, part of a protective behavior in response to noxious cutaneous stimuli.

While both of these circuits can be measured in humans, current techniques fail to give an accurate picture of their operation. In humans, sEMG recordings provide low-resolution data and underestimate the duration of both circuits (*6*–*8*). On the other hand, iEMG using fine wires or needles provides single-unit resolution and a better estimation of inhibition duration but is invasive and only samples one or few MUs within highly heterogeneous motoneuron populations and therefore falls short in estimating the temporal characteristics of circuits as a whole. To overcome the limited sampling, participant data are usually pooled (*9*), but doing so relies on the assumption that motor pool properties are similar between individuals (*10*), which we know is not the case (*10*, *11*). That is, only limited mechanistic, physiological insights into the operation of spinal circuits can be gained using sEMG and iEMG methods.

If, as in animal studies (*12*), the function of human inhibitory circuits reflects the status of a disease such as amyotrophic lateral sclerosis, then it will be necessary to measure inhibition accurately, reproducibly, and in a participant-specific manner. Recent studies have pointed to spinal circuit alterations in neurological diseases (*13*–*15*), yet the technical constraints imposed by traditional sEMG and iEMG have meant the loss of participant specificity. Enhanced sampling methods capable of sampling multiple MUs and capturing their respective spinal cord circuit features will greatly improve the accuracy of physiological and pathophysiological measures. We have therefore sought to establish methodologies for probing spinal circuit function at higher resolution, which would enable more precise assessments of MU behavior and individual variability.

Advancements in high-density sEMG (HDsEMG) techniques are transforming the landscape in the field of motor physiology (*16*). This noninvasive method uses arrays of closely spaced electrodes to achieve high spatial and temporal resolution of the activity of individual MUs, recording a high proportion of units that comprise an individual muscle. HDsEMG has facilitated the study of a variety of MU properties, such as conduction velocity and discharge rate (*17*), as well as network-level features such as common synaptic inputs to motoneurons during movements, indicating a potential to shed light into the mechanisms of motoneuron integration of input and recruitment across a pool (*18*, *19*).

In this work, we present an optimized framework for the use of HDsEMG in the study of spinal circuits in both upper and lower limb muscles. We focus on two distinct inhibitory pathways, CSP and reciprocal inhibition, as these circuits are involved in key physiological functions and have biomarker potential (*13*, *20*, *21*). Using simultaneous testing across different muscles, we draw inferences about distinct inhibitory spinal pathways. By successfully sampling multiple MUs per participant, we were able to conduct participant-specific analyses of the temporal properties of CSP and reciprocal inhibition. This allowed us to characterize these circuits by taking participant-level variations into account and to provide more accurate estimates of functional inhibition acting on firing motoneurons. We then modeled these circuits in silico to obtain insights into the relationship between synaptic inhibition and motoneuron intrinsic properties. Together, we demonstrate how HDsEMG can be used to investigate spinal cord circuit mechanisms and support its broader application in neuromuscular research and clinics.

## RESULTS

### Participant-specific analysis of CSP

Reasoning that the use of HDsEMG provides an opportunity to study stimulus-evoked inhibition of many motoneurons simultaneously and that the duration of inhibition reflects the temporal profile of the inhibitory input received by each motoneuron (*7*, *22*), we turned to methods of quantifying the times of onset and termination of inhibition based on spiking profiles. Peristimulus time histograms (PSTHs) constructed from single unit data show spiking activity in response to the stimulus and accurately reflect the onset latency of inhibition of a MU (*23*, *24*). Plotting the frequency of firing, peristimulus frequencygrams (PSFs) can pinpoint when the firing frequency returns to its baseline (prestimulus) level, reflecting the termination of inhibition of firing (*7*, *13*, *22*). Thus, from these two plots, the duration of inhibition during ongoing MU activity can be calculated (*7*, *22*, *25*). We then used PSTH (1-ms bins typically containing one to two MU firings) and PSF measures for all units recorded via HDsEMG, including all MUs with robust inhibition defined as a deviation of the cumulative summation (CUSUM) exceeding the limits [i.e., error-box (*26*)] seen in the 200 ms preceding the stimulus (fig. S1). CUSUM provides a sequential monitoring of the deviations from the mean MU firing occurrence (for PSTH) or mean firing rate (for PSF) from a prestimulus baseline window, with deflections of the CUSUM trace providing a sensitive approach toward the objective identification of the onset and duration of inhibition on a unit-by-unit basis. In our measurements, we are capturing MU discharge during steady voluntary contraction and determining the duration of the period in which MU firing rate is affected by the inhibitory synaptic input—a parameter we term “functional inhibition,” recognizing that this does not reflect an inhibitory postsynaptic potential (IPSP) alone. That is, functional inhibition refers to a stimulus-evoked reduction in motoneuron firing output during voluntary contraction, reflecting the duration of inhibition as changes in discharge probability and firing rate.

**Fig. 1.**
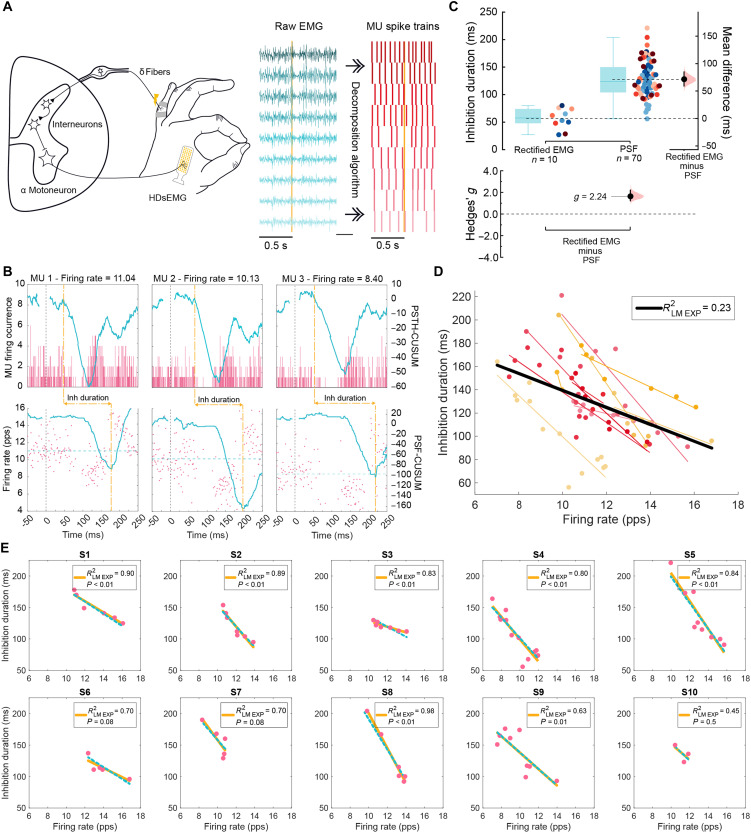
Characterization of CSP on individual motoneurons in humans. (**A**) Schematic of the CSP spinal pathway and experimental setup (left) and the decomposition methods used to extract the firing phase and frequency of individual MUs with HDsEMG (right). (**B**) Examples of PSTH (top) and PSF (bottom) of individual MUs from the FDI muscle active at different frequencies, with CUSUM traces in blue for each. Dotted yellow lines depicted inhibition start defined by PSTH and end by PSF. (**C**) Estimation plots illustrating the comparison of the inhibition duration estimation methods: rectified sEMG and individual MUs with the PSF method, with dots color-coded per individual. (**D**) Estimation of inhibition duration of single MUs and their firing rate across participants. Colored dots represent MUs and the linear regression fit per individual; black line represents the linear regression fit for the group data with respective *R*^2^ shown. (**E**) Inhibition duration of single MUs versus their firing rate per individual (pink dots). Yellow lines represent the individualized linear regression models (LM_exp_) with respective *R*^2^ and *P* value; blue dotted lines represent the LMM regression per participant; pps, pulse per second.

We initially focused on the upper limb CSP, a robust and reproducible stimulus carried by A-delta fibers to spinal dorsal interneurons, which in turn inhibit motoneurons innervating intrinsic hand muscles (Fig. 1A) (*5*, *27*–*29*). We placed 64-channel HDsEMG grids on top of the first dorsal interosseous (FDI) muscle and instructed the participants to perform a pinching task for 200 s at 10% of their maximum voluntary contraction (MVC), electrically stimulating the fifth finger [at ×10 the sensory threshold (ST)] every 1.8 s (*28*). Single MUs were decomposed from the raw EMG (Fig. 1A) to measure the temporal properties of inhibition (Fig. 1B).

In addition to the decomposition of individual MUs, the HDsEMG grid can also act as a standard bipolar sEMG electrode by recording the voltage difference between two adjacent electrodes, providing a conventional sEMG signal for assessing overall muscle activity (fig. S1). We therefore compared the duration of inhibition obtained from the individual MUs decomposed with HDsEMG to the conventional sEMG signal obtained during the same recordings (Fig. 1C). We found a consistently shorter CSP duration when measured from the rectified sEMG (58 ± 18 ms) compared to the durations measured from individual MUs using PSF (121 ± 22 ms), indicating that bipolar sEMG recordings are sensitive only to the shortest duration inhibitory responses (μ_diff_ = 72 ms, 95% confidence interval (CI) = [59, 85]; *g* = 2.25, 95% CI = [1.82, 2.84]). The sEMG provides an averaged signal of multiple MUs, each with slightly different timing for inhibition onset and termination. Because sEMG estimation of inhibition is defined solely by spike occurrence or spike count (*7*), MUs with shorter inhibition periods resume firing earlier, potentially masking ongoing inhibition in other units that experience longer duration inhibition. Moreover, even when an MU resumes firing, its discharge rate may remain below baseline reflecting a continued hyperpolarization of the membrane potential. As a result, PSF estimates taken from individual MUs recorded with HDsEMG better reflect the underlying membrane dynamics and thus capture longer inhibition durations by incorporating both the presence and amplitude of the inhibitory postsynaptic potentials (*30*). Overall, single-unit HDsEMG data revealed a wide range of durations, with many units being inhibited over twice as long as estimated by rectified global sEMG.

The duration of the CSP during voluntary contractions is highly variable and influenced by the MU discharge rate (*6*, *20*). Therefore, interpreting inhibition duration is best done relative to firing rate (*6*, *20*). The coefficient of determination (*R*^2^) is a standard metric for assessing goodness of fit (*31*, *32*) and is widely used to validate linear relationships, such as firing rate versus inhibition duration in motoneuron studies (*7*, *13*, *14*, *20*). An *R*^2^ value of 1 indicates a perfect linear relationship, meaning that firing rate fully accounts for the variability in inhibition duration, whereas an *R*^2^ of 0 means that firing rate provides no explanatory power, with inhibition duration varying independently. For this study, we classified the strength of the *R*^2^ using thresholds of 0.67, 0.33, or 0.19, corresponding to strong, moderate, and weak relationships, respectively, based on established statistical conventions (fig. S2) (*33*, *34*). In addition, we report *P* values to assess whether the relationship between firing rate and inhibition duration is statistically significant.

When plotting firing rate against CSP duration, we observed that inhibition duration is poorly predicted by firing rate for all MUs combined from all participants, as shown by a weak *R*^2^ (Fig. 1D). However, this *R*^2^ (*R*^2^ = 0.24) was similar to that reported previously using iEMG for healthy participants (*R*^2^ = 0.31) (*13*). Although low *R*^2^ values may still help identify trends within the data, they are limited in predictive accuracy and precision (*35*). FDI MUs showed a significant negative relationship between recruitment threshold and firing rate, supporting the expected hierarchical organization of motoneuron activation under our recording conditions (fig. S7A and table S17).

While single-unit iEMG has been useful to establish group-level clinical outcomes when pooling data from groups of participants (*13*, *14*), it typically samples only one to two MUs per participant in studies of inhibitory spinal microcircuits (*13*, *14*). This limited sampling of the motor pool within a single individual fails to capture motoneuron heterogeneity within and between participants, resulting in low-dimensional data that reduce measurement precision and limit the ability to make participant-specific assessments (*9*). In contrast, the increased MU sampling enabled by HDsEMG allows for the creation of robust hierarchical datasets (level 1, MUs; level 2, participants), making it possible to assess how individual differences influence the relationship between inhibition duration and discharge rate for CSP.

To quantify the influence of participant-specific variability in the interpretation of CSP experimental data obtained with HDsEMG, we implemented a linear model (LM_exp_) for each individual and used a linear mixed model (LMM) to account for participant-specific random effects. For the FDI muscle, individual LM_exp_ consistently outperformed the pooled correlation for firing rate versus inhibition duration relationships, yielding higher *R*^2^ values. Specifically, 6 of the 10 participants exhibited strong *R*^2^, indicating that firing rate explained a substantial portion of the variability in inhibition duration (*P* < 0.05). An additional two participants had a high *R*^2^ but *P* = 0.08, potentially reflecting low power due to the few MUs extracted. The remaining two individuals exhibited moderate *R*^2^ with the participant with the fewest sampled MUs (three MUs) showing no meaningful relationship (*P* = 0.5) (Fig. 1E). The LMM results indicate that inhibition duration varied significantly with firing rate, with a decrease of 15 ms in inhibition duration for every 1-Hz increase in discharge rate (table S1). However, the LMM captured 84% of data variability (*R*^2^ = 0.84), driven by intersubject differences that accounted for 80% of total variance [intraclass correlation coefficient (ICC) = 0.80]. The LMM analysis revealed significant differences in inhibitory profiles across participants, with individuals with longer inhibition durations having less reduction in inhibition duration with increasing firing rate (see table S1). The disparities in the measured effects between grouped and participant-specific analyses underscore the importance of considering individuality when interpreting the effect of synaptic inhibition on MU behavior.

Together, we demonstrate that HDsEMG is a more sensitive method than traditional EMG approaches for estimating CSP duration. Unlike traditional methods, HDsEMG offers a noninvasive estimation comparable to single-unit iEMG while also allowing for the sampling of multiple MUs per participant. Furthermore, the hierarchical datasets generated through HDsEMG provide a robust framework for studying inhibition duration on a participant-specific level, accounting for interindividual variability.

### Predicting CSP characteristics using in silico modeling

Inhibition of motoneurons depends on the strength of the inhibitory inputs and the response properties of the motoneurons. Intrinsic neuronal properties influence how synaptic inputs are integrated, potentially affecting inhibition duration. For example, the physical dimensions of motoneurons are directly linked to their intrinsic properties: Motoneurons with larger somas and dendritic trees require greater synaptic input to achieve the same membrane voltage change as smaller motoneurons (*36*, *37*).

Historically, in vivo and in vitro preparations from animals have enabled single-cell recordings, allowing for controlled current injections and intracellular voltage measurements (*38*–*41*). These techniques provide insight into synaptic inputs and intrinsic properties, such as whole-cell capacitance, which can be used as a surrogate marker of motoneuron size. HDsEMG, however, cannot directly measure these parameters.

During voluntary contractions, FDI motoneurons firing at higher firing rates exhibit shorter CSP inhibition despite delivering the same nerve stimulation intensity, indicating that motoneurons likely receive the same synaptic drive from afferents (*36*, *37*, *42*). Is this effect purely due to firing rate, and/or do differences in intrinsic properties play a role? To disentangle these factors, we built an in silico model replicating FDI CSP inhibition and manipulated motoneuron size and firing rate to assess their distinct effects on inhibition duration.

**Fig. 2.**
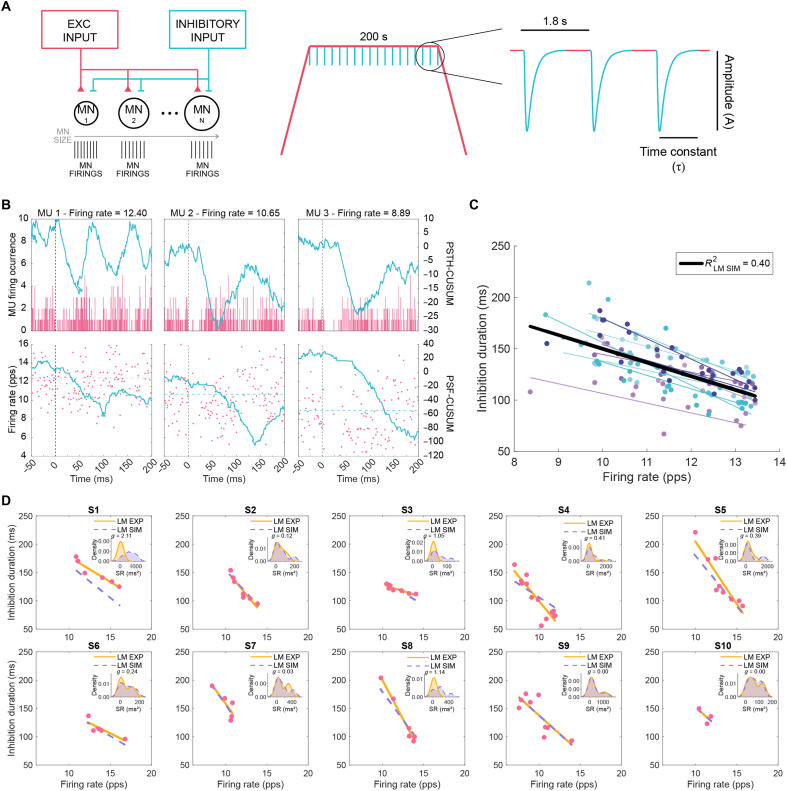
Characterization of CSP on individual motoneurons with computational modeling. (**A**) Schematic of the in silico computational model depicting the common excitatory input and inhibitory input delivered to motoneurons (MN) of varying size (left), used to simulate the experimental MU firing during voluntary contraction with periodic inhibitory drive to motoneurons delivered during the plateau phase (right). (**B**) Examples of PSTH (top) and PSF (bottom) of simulated MUs at different frequencies, with CUSUM traces in blue. (**C**) Estimation of inhibition duration of single MUs and their firing rate across the LM_sim_ optimized for each participant. Individual simulated MUs and each of the LM_sim_ fits are shown in color; black line represents the linear regression fit for the simulated group data with respective *R*^2^ shown. (**D**) Experimental MU firing rate and inhibition duration (pink dots) with respective regression line (orange line; same LM_exp_ as in Fig. 1E) and optimal LM_sim_ fit for each participant (dotted purple line). Inset plots show distributions of square of residuals (SR) as kernel density estimates along with the corresponding Hedges’ *g* value.

We generated a two-compartment Hodgkin-Huxley biophysical model (*43*) and simulated the CSP experiments previously described. The model simulated common input to a motor pool of 20 motoneurons replicating an isometric contraction, with an inhibitory synaptic input delivered every 1.8 s during the steady phase of the isometric contraction (Fig. 2A). The motoneurons were modeled on the basis of the characteristics of S-type motoneurons derived from in vivo motoneuron recordings from cats (*44*–*49*), which primarily govern sustained, low-force contractions (table S6). These motoneurons were assigned sizes ranging from the smallest to the largest (189 to 214 pF of cell soma capacitance), with properties linearly interpolated to reflect a physiological gradient in excitability and recruitment thresholds, consistent with known motor pool organization (*36*, *50*, *51*). We recorded the spike train outputs in all motoneurons across 154 realizations, each defined by a unique combination of amplitude [A; 1 to 3 arbitrary units (a.u.), in 0.2-a.u. steps] and tau (τ; 7 to 20 ms, in 1-ms steps) of the inhibitory synaptic input, a parameter analogous to an inhibitory postsynaptic current (IPSC), distributed uniformly across motoneurons. For each realization, we quantified the duration of functional inhibition of each simulated motoneuron using the same PSTH and PSF methods applied to the experimental HDsEMG data (Fig. 2B) and kept only the units showing inhibitory responses that exceeded the variability of the 200-ms prestimulus baseline based on the CUSUM approach described above for HDsEMG recordings (fig. S1). The simulated inhibition durations and respective firing rates were linearly fitted for each realization, leading to 154 linear models (LM_sim_) with a range of *R*^2^, slopes and intercept consistent with those observed across participants (fig. S3A).

We then identified the LM_sim_ best suited to replicate the experimentally observed dependency of inhibition duration on firing rate for each of the 10 participants. We inputted each participant’s experimental firing rates into all 154 LM_sim_ formulas and selected the LM_sim_ with the lowest mean squared error (MSE), which minimized errors between predicted and observed inhibition durations. The LM_sim_ with the lowest MSE was selected as the best-fitting model for that participant, ensuring minimal prediction error at the individual level (figs. S2D and S3B and table S2).

To illustrate comparisons between experimental data fits (LM_exp_) and the LM_sim_ optimized for each of the 10 participants (Fig. 2C), we analyzed the distributions of square of residuals between the fits relative to each of the experimental data points and calculated Hedges *g′* to assess differences between the distributions (Fig. 2D and fig. S3C). Apart from participants 1, 3, and 8 who showed a notable discrepancy between the residuals, effect sizes for the remaining participants were small (0.15 < *g* < 0.50) or negligible (*g* < 0.15). These results suggest that, while the computational model generally captured observed CSP inhibition duration dependency on firing rate, its performance varied across individuals.

### Motoneuron size does not contribute to variations in inhibition duration according to biophysical modeling

Motoneurons vary in size and form a diverse group, even within the same motor pool. This structural variation leads to a graded recruitment pattern in response to common input, where smaller, low-threshold motoneurons reach their action potential threshold before larger ones. As the input increases, smaller motoneurons will fire faster at the time that the larger motoneurons are recruited (and fire at low rates). Our experimental and simulated results indicate that the duration of functional inhibition is longer in later recruited motoneurons, which fire slower (toward the left-end of the regression panels). Yet, it is unclear why the inhibition is longer in these neurons: Is it because they are larger in size (late recruited motoneurons are usually larger motoneurons) or because they fire at a lower rate (i.e., receiving lower net excitatory input which may cancel out inhibition)? We cannot answer this question experimentally, so we used our in silico model to investigate whether motoneuron size influences variations in inhibition duration.

**Fig. 3.**
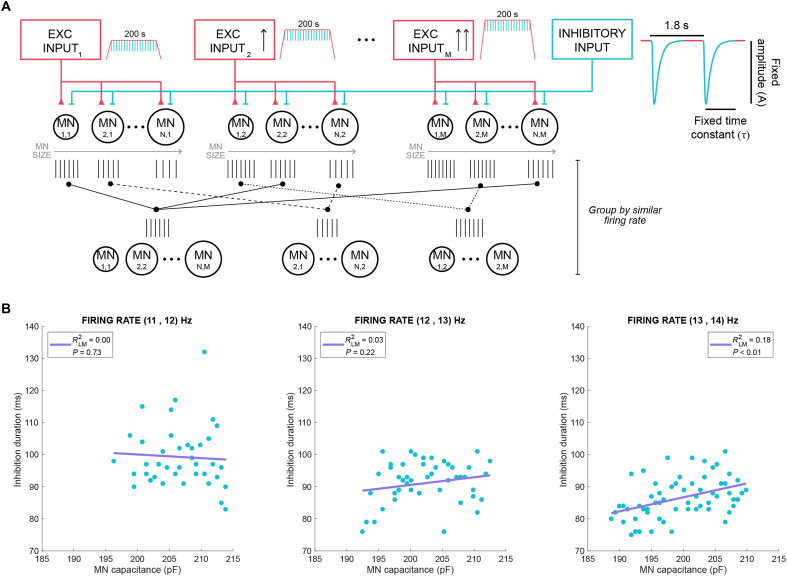
Influence of motoneuron size on inhibition duration in silico. (**A**) Schematic of the computational model, showing the different common inputs that generated three distinct firing rates, with MN size varying per discharge rate group and with inhibition strength of fixed amplitude and duration. (**B**) Inhibition duration across simulated motoneurons with varying size (blue dots) with each plot representing a different firing rate, with purple line representing the linear regression between capacitance and inhibition duration. Capacitance values represent simulated soma capacitance. Simulations were obtained using optimized parameters for participant 8 (inhibitory input amplitude of 2.8 a.u. and duration of 19 ms).

We simulated motoneurons using the optimized model parameters from participant 8, characterized by long-tau (19 ms) and high-amplitude (2.8 a.u.) simulated IPSC that produced a strong dependency of functional inhibition with firing rate for the simulated MUs (*R*^2^ = 0.92, *P* = 2.37 × 10^−07^; table S2). We also ran a secondary set of simulations using parameters from participant 4, which differed substantially in both the optimal parameter values for synaptic input amplitude (1.8 a.u.) and tau (7 ms) and the linear fit (*R*^2^ = 0.37, *P* = 0.0269, table S2). These contrasting conditions provided an opportunity to explore the effects of motoneuron size under distinctly different simulation conditions. For these simulations, we doubled the simulated motoneuron pool (40 instead of 20) to improve resolution in characterizing intrinsic property gradients. The common input was calibrated to generate steady firing rates of 11 to14 Hz during the plateau phase of the ramp (0.2 to 0.28 a.u. in 0.01-a.u. steps). Synaptic inputs, fixed in amplitude (1.8 or 2.8 a.u.) and duration (7 or 19 ms) to reflect physiological sensory spinal circuit behaviors (*12*, *42*, *52*), were delivered every 1.8 s, while motoneuron size was systematically varied on the basis of S-type motoneuron parameters (table S6), as previously described the CSP (Fig. 3A). This approach enabled precise control of MU firing rates and manipulation of motoneuron dimensions within set ranges, isolating the effect of cell size (i.e., capacitance) on inhibition duration.

To examine the effect of motoneuron size across different discharge rates, motoneurons were clustered into three different groups by firing rate (11 to 12, 13 to 14, and 14 to 15 Hz) and compared across soma sizes (Fig. 3B and fig. S4). Group comparisons revealed that inhibition duration decreases as the firing rate increases (table S3). However, within each firing rate group, motoneuron capacitance did not explain variations in inhibition duration as shown by the very weak *R*^2^ values. These findings were similar for the two sets of optimizing parameters tested for A and τ (Fig. 3B and fig. S4). Also, motoneuron capacitance did not introduce any variability to the relationship between inhibition duration and firing rate as shown by the negligible ICC (ICC_capacitance_ < 0.20; table S3).

These results indicate that, under the controlled conditions of our in silico model, and for simulated S-type motoneurons, the duration of functional inhibition is more strongly influenced by motoneuron firing rate than by size. This suggests that active properties (such as firing rate) may play a greater role than passive membrane properties (such as capacitance) in determining functional inhibition.

### Estimating reciprocal inhibition in the tibialis anterior with HDsEMG

**Fig. 4.**
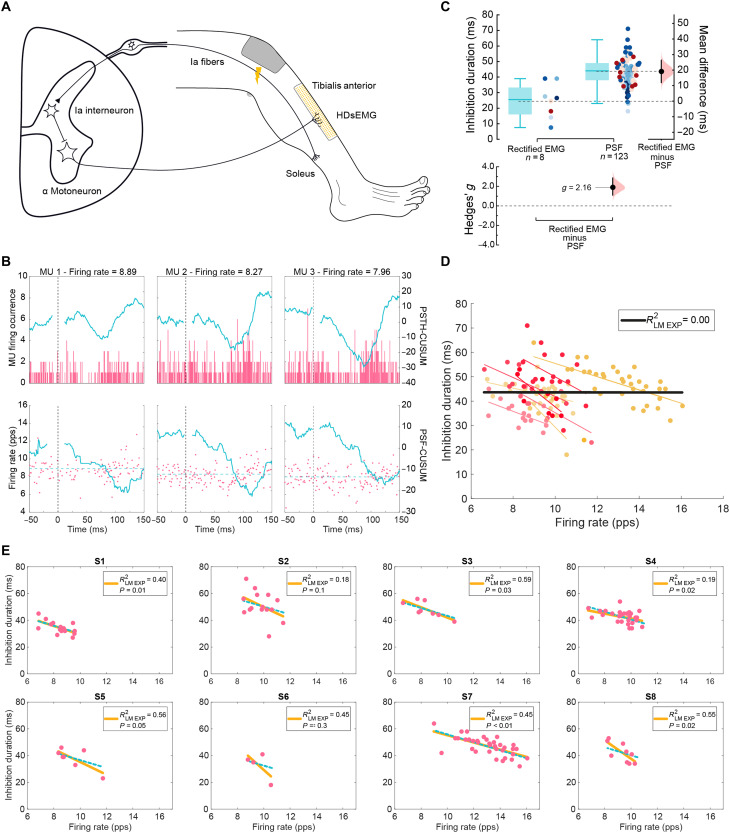
Characterization of reciprocal inhibition of the TA on individual motoneurons in humans. (**A**) Schematic of the reciprocal inhibition spinal circuit and experimental setup used to measure inhibition from the TA muscle. (**B**) Examples of PSTH (top) and PSF (bottom) of individual MUs at different frequencies following TA muscle activation, with CUSUM traces in blue for each. (**C**) Estimation plots depicting comparison of the inhibition duration estimation methods: rectified sEMG and individual MUs with the PSTH-PSF method. (**D**) Estimation of inhibition duration of single MUs with varying firing rate across participants. Colored dots represent MUs and the linear regression fit per individual; black line represents the linear regression fit for the group data with respective *R*^2^ shown. (**E**) Inhibition duration of single MUs and their firing rate per individual (pink dots). Yellow lines represent the individualized linear fits (LM_exp_) with respective *R*^2^ and *P* value; blue dotted lines represent the LMM regression per participant.

To examine whether our finding that inhibition depends mainly on firing rate can be extrapolated to other inhibitory circuits, we next turned to the lower limb, with a focus on a different inhibitory circuit—reciprocal inhibition. This circuit involves recruitment of a different set (compared to CSP) of sensory afferents (group I) and spinal interneurons (Ia interneurons) and is responsible for flexor-extensor alternations during movement (*8*, *53*). To study reciprocal inhibition, we tested eight individuals by placing a 256-multielectrode HDsEMG grid over the tibialis anterior (TA) muscle and evoked reciprocal inhibition from triceps surae afferents to TA motoneurons through tibial nerve stimulation (Fig. 4A). The temporal properties of inhibition were estimated as above using PSF and PSTH (Fig. 4B).

We first compared the estimates of inhibition duration between the individual MUs decomposed through HDsEMG and the conventional sEMG signal obtained during the same recordings obtained at 10% MVC for ankle dorsiflexion. Similar to our findings for CSP, sEMG measurements underestimated the duration of reciprocal inhibition (25 ± 11 ms), compared to the inhibition of individual MUs (42 ± 7 ms) obtained using HDsEMG (μ_diff_ = 19 ms, 95% CI = [12, 26]; *g* = 2.16, 95% CI = [1.35, 3.13]) (Fig. 4C). This again highlights that individual MUs sampled with HDsEMG provide a wider range of inhibition durations.

Given that inhibition duration varies with discharge rate for CSP, we examined the dependence of reciprocal inhibition on motoneuron firing rates. Pooled MU data across participants showed that inhibition duration is poorly predicted by firing rate when aggregating MU data from multiple participants (*R*^2^ = 0.00; Fig. 4D). On the other hand, individual LM_exp_ yielded a moderate *R*^2^ for four of the eight participants (1, 3, 4, and 8; *P* < 0.05). For another participant, borderline-weak *R*^2^ was observed (*R*^2^ = 0.19, *P* = 0.02). Participant 5 had a moderate *R*^2^ but a *P* = 0.05, whereas participants 2 and 6 did not have a statistically significant relationship. An LMM with random intercept revealed a decrease of 3 ms in inhibition duration for every 1-Hz increase in discharge rate, with intersubject variability reflected by an *R*^2^ of 0.56 and an ICC_subject_ of 0.60 (table S4). These results highlight the importance of accounting for intersubject variability to accurately interpret the relationship between discharge rate and duration of reciprocal inhibition.

Overall, individual LM_exp_ suggest a weaker relationship between reciprocal inhibition duration of TA MUs and discharge rate compared to that seen in CSP duration in FDI MUs. Although we sampled an average of 15 MUs for reciprocal inhibition and 7 MUs for CSP, firing rate tended to exhibit less explanatory power for reciprocal inhibition LM_exp_ (four moderate and one weak *R*^2^; *P* < 0.05) than for CSP LM_exp_ (six strong and one moderate *R*^2^; *P* < 0.05). For CSP, individuals with fewer sampled units generally exhibited lower *R*^2^ and higher *P* values (e.g., participant 10; Fig. 1D). However, this pattern was not observed for reciprocal inhibition, where some participants with many sampled units still showed a weak *R*^2^ and, in some cases, no statistically significant linear relationship (e.g., participant 2, Fig. 4D).

Reciprocal inhibition measured from the TA had an ~fourfold shorter inhibition duration and a fivefold smaller change in inhibition duration with discharge rate than the CSP for FDI (tables S1 and S4). This could affect the relationship between firing rate and inhibition duration by reducing the overall influence of inhibition on motoneuron discharge characteristics, potentially contributing to a weaker correlation between reciprocal inhibition duration and firing rate in TA MUs compared to CSP duration in FDI MUs. Nevertheless, similar to the experimental observations for the CSP, our results highlight the need to sample multiple units per participant to capture individual reciprocal inhibition effects. We also extracted recruitment thresholds from the initial ramp phase in seven of the eight participants. As with the FDI, we observed a significant negative relationship between recruitment threshold and firing rate in the TA, consistent with a size principle–based recruitment pattern (fig. S7B and table S18).

### Predicting reciprocal inhibition characteristics using in silico modeling

**Fig. 5.**
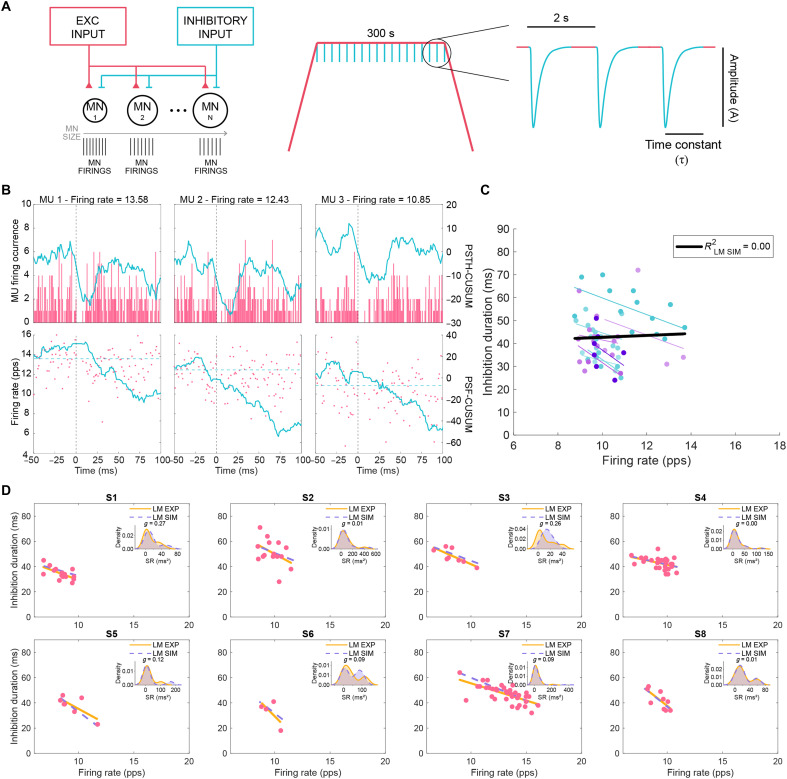
Characterization of reciprocal inhibition on individual motoneurons with computational modeling. (**A**) Schematic of the in silico computational model used to simulate the common input and inhibitory input delivered to MN of varying size (left), during artificial voluntary contraction with periodic inhibitory inputs to motoneurons delivered during the plateau phase (right). (**B**) Examples of PSTH (top) and PSF (bottom) of simulated MUs at different frequencies, with CUSUM traces in blue. (**C**) Simulated data points and respective linear fits for inhibition duration relationship with discharge rate across the LM_sim_ optimized for each participant (different shades of blue per optimized LM_sim_); black line represents the linear fit for the simulated group data with respective *R*^2^ shown. (**D**) Experimental MU inhibition durations and firing rate (pink dots) with respective regression line (orange line; same LM_exp_ as in Fig. 4E) and optimal LM_sim_ fit for each participant (dotted purple line). Inset plots show distributions of SR as kernel density estimates and the corresponding Hedges’ *g* value.

We now extended our in silico approach to investigate reciprocal inhibition. We simulated the common input into a motor pool of 20 motoneurons of varying size (same as for CSP; table S6) to reproduce an isometric contraction. Initially, we ran simulations with a 0.20-a.u. common input, which replicated experimental firing rates. However, to minimize background noise that affected PSTH-PSF inhibition duration estimates for some realizations, we performed additional simulations with a reduced 0.15-a.u. common input. This adjustment preserved realistic firing rates while reducing noise interference when estimating inhibition duration. Inhibitory synaptic inputs received by motoneurons during the steady-state phase of the isometric contractions were delivered every 2 s (Fig. 5A). Motoneuron size was systematically varied using the same parameters employed in earlier simulations (table S6). We recorded spike discharges across realizations, each defined by a combination of inhibitory inputs of varying A (1 to 3 a.u. in 0.2 steps) and τ (1 to 4 ms in 1-ms steps). We analyzed inhibition duration of each simulated motoneuron using the PSTH and PSF, keeping only units with detectable inhibition (Fig. 5B). The simulated firing rates and inhibition durations were fitted per realization (fig. S5). For some realizations, we obtained two or fewer units with detectable inhibition, which did not allow us to establish linear regressions. Across all A and τ combinations, with either 0.20 or 0.15 a.u. common input, *R*^2^, slope, and intercept showed no consistent pattern (fig. S5A). We then chose the LM_sim_ that best explained the experimentally observed dependency of inhibition on firing rate for each of the eight individuals, as previously done for the CSP simulations (figs. S2D and S5C and table S5).

When comparing the optimized LM_sim_ for each participant (Fig. 5C) to the experimental fits (LM_exp_; Fig. 5D) based on distributions of square of residuals between experimental data points and fitted lines, we observed only small effect sizes for participants 1 and 3, while no effects were found for the other participants. Although the LM_sim_ realizations did not follow a consistent selection pattern and appeared to be nearly random (fig. S5, C and D), they nonetheless produced fits that qualitatively resembled the experimental reciprocal inhibition data. However, the generally low *R*^2^ values indicate that these fits may not reflect a strong or systematic relationship. While the biophysical models yielded fits that occasionally matched the experimental data, these matches may reflect partial or incidental agreement.

## DISCUSSION

In this work, we used HDsEMG to study the temporal properties of the CSP in a hand muscle (FDI) and reciprocal Ia inhibition in the lower leg (TA muscle). We successfully sampled multiple MUs per individual and demonstrated that functional inhibition duration is best interpreted in the context of varying MU discharge rates, emphasizing the need for participant-specific analysis. By measuring inhibition using firing rates, we are not measuring IPSP but rather how the inhibition affects motoneuron output. For example, large amplitude IPSPs may appear to be of shorter duration if they recruit active currents [e.g., hyperpolarization-activated current (I_h_) (*54*, *55*) or CaV3 currents (*56*, *57*)], leading to earlier onset firing. We also used in silico modeling, which indicated that the underlying firing rates and characteristics of the inhibitory inputs rather than variations in S-type motoneuron size play roles in determining the functional duration of inhibition across different MUs.

### Participant-specific analysis of spinal inhibitory circuits

Interindividual variability is intrinsic to the human nervous system, as evidenced by neuroimaging (*58*), electroencephalography (*58*), transcranial magnetic stimulation (*59*, *60*), and electromyographic and kinematic studies (*10*, *61*, *62*). Such variability influences computational modeling of human neurophysiology (*10*, *63*–*65*). In clinical neurophysiology, traditional methods such as sEMG or iEMG yield low-dimensional data due to their technical limitations, which limit the assessment of interindividual differences. For example, the electrical response obtained with sEMG does not inform about individual MUs, and single MU iEMG is invasive, leading to sampling of only a few units (*9*). Such constraints result in data homogeneity being assumed a priori, leading to individual MU data being aggregated across participants and analyzed collectively. In contrast, with HDsEMG, we were able to extract multiple units per individual, resulting in datasets with a hierarchical structure: that is, MU data are nested within participants. This not only opens the possibility for clinically relevant participant-specific studies with HDsEMG but also introduces more rigor in data interpretation by addressing data dependencies, thus reducing the risk of false positives (*66*–*68*).

For example, recent work using iEMG established weak relationships (*R*^2^ < 0.33) between CSP inhibition duration and MU discharge rate (*R*^2^ = 0.30) (*13*), similar to our findings when we plotted all sampled MUs together (*R*^2^ = 0.24). However, by sampling an average of seven units per individual, unlike for iEMG (*13*), we were able to establish linear regressions for each individual and use more rigorous statistical approaches that account for the high (80%) interindividual variability of the CSP. Together, our findings highlight the potential of HDsEMG in the study of inhibitory spinal circuits by permitting participant-specific clinical insights.

### CSP and reciprocal inhibition: Methodological and physiological considerations

We observed that interparticipant variability, the strength of inhibition, and the relationships between discharge rate and inhibition duration were not the same for the CSP measured from the FDI and reciprocal inhibition recorded from the TA muscle. These differences could be influenced by multiple factors such as different stimulation paradigms to evoke CSP and reciprocal inhibition (*22*), upper and lower limb motoneurons, and microcircuit physiologies and contraction profiles.

For the CSP, we used a stimulation protocol designed to robustly activate A-delta fibers mediating the withdrawal reflex (*5**,* *27*–*29*). To evoke reciprocal inhibition, we stimulated the tibial nerve at 1.1× the minimum H-reflex amplitude threshold to minimize activation of afferents other than the largest (e.g., proprioceptive Ia afferents) and cross-talk contamination from heteronymous monosynaptic Ia afferent excitation to the TA muscle (*69*, *70*). Therefore our stimulation protocol likely recruited robust Ia reciprocal inhibition. The synaptic input received by motoneurons when evoking the CSP is considerably slower (*71*) likely because of γ-aminobutyric acid type A receptor activation (*72*), whereas reciprocal inhibition is purely glycinergic (*73*, *74*); such factors could lead to differences between CSP and reciprocal inhibition in the strength and timing of inhibition in motoneurons (*75*, *76*). Furthermore, although TA and FDI muscles have similar muscle fiber composition, contractile properties, and discharge rates (*77*), physiological differences may exist between upper and lower limb circuitries that may affect the strength of the correlations with discharge rate. One must also consider the impact of active conductances in our PSTH-PSF estimations; for example, computational studies have suggested that activation of I_h_ at more hyperpolarized voltages, which may have been elicited at stronger stimulation intensities may produce a faster rebound that could shorten inhibition (*55*). Last, one must consider that synaptic noise introduces variability in spike timing, which can disproportionately affect PSF and PSTH estimates, particularly when detecting smaller-size functional inhibition (*22*, *78*).

### Accuracy of in silico modeling of inhibitory spinal circuits

Biophysical simulations for the CSP revealed that *R*^2^ increased with higher inhibition amplitude (A) and longer tau (τ). The optimized parameters for each participant varied widely across participants (Fig. 2D and fig. S3B). Some individuals, particularly those near the limits of the hyperparameter space, showed poor fits (e.g., participant 1), while others had a better fit to the experimental HDsEMG data (e.g., participant 5). In some cases, multiple realizations could perform similarly (participant 2) and, for others, there were fewer realizations with low MSE (participant 6; Fig. 2D and fig. S3, B and C). Together, these results highlight the complexity of simulating biological relationships for spinal microcircuits with robust inhibition.

Caution must be taken about the interpretation of the robustness and predictive power of the in silico models. The HDsEMG data for reciprocal inhibition did not yield any strong *R*^2^ coefficients for the tested individuals (Fig. 4D), and simulating data reproducing experimental relationships with weak *R*^2^ values is inherently more challenging (*79*). The optimized parameters for most participants (six of eight) were located near the boundaries of the explored hyperparameter space, both near the lower and upper ends of A and τ (fig. S5, C to D), suggesting that our simulations may not have fully captured the range of physiological variability. While simulations were more realistic and performed well for circuits with robust inhibition such as the CSP (fig. S3A), they were less reliable for pathways with shorter inhibition such as reciprocal inhibition (fig. S5, A and B). In these cases, multiple factors, such as synaptic noise or fewer inhibited spikes, may influence the linear relationships between inhibition duration and MU discharge rate, potentially leading to unrealistic and oversimplified fits that happened to align with the experimental data (*80*). These findings suggest that reciprocal inhibition measured in our experimental conditions, is not only physiologically weaker than CSP but also more difficult to quantify and model accurately, thus stressing the need for caution when working with weak or noisy fits in both experimental and simulated HDsEMG data.

Motoneurons are complex neuronal cells characterized by an extensive dendritic tree, large soma, complex axonal excitability features, high density and variety of synaptic inputs, and diverse, spatially localized ion channels and metabotropic receptors (*81*). Such complexity would be best captured with multicompartment models, but, for our purposes, we used a simple two-compartment model as it has been shown that both single- and two-compartment models can successfully replicate variations in MU firing under different modulatory and synaptic inputs (*10*, *82*–*84*). Our model used simplified ionic conductance parameters, omitting additional active conductances (e.g., persistent inward currents) that could potentially influence the simulated MU firing rates. Furthermore, we did not include any neuromodulatory paramaters; such effects would affect intrinsic motoneuron properties as well as synaptic inputs, which may affect inhibition duration (*85*). Despite these constraints, our in silico simulations successfully reproduced the observed MU firing rate-inhibition duration relationships across most participants, indicating that synaptic input dynamics primarily drive these phenomena.

In line with recent work (*10*), we emphasize the importance of participant-specific parameter considerations when generating computational models for the study of spinal circuits. Biophysical models accounting for individual participants not only better replicate experimental findings but are also essential for understanding spinal connectivity and functional impact of synaptic inputs. This is because direct, invasive measurements are not feasible in humans (*80*), thus making in silico models indispensable for studying human spinal synaptic physiology.

### Dependence of inhibition duration on firing rate and motoneuron size

Our computational model was fine-tuned on experimental data obtained at 10% MVC, with simulations reflecting the behavior of early-recruited MU, likely representing a portion of small-sized motoneurons within the FDI muscle [the “size principle” (*36*, *42*)]. The differences in soma capacitance between the largest and smallest motoneurons in our simulated dataset did not exceed 15%. However, incorporating parameters from fast motoneurons typically recruited at higher MVCs could reveal ~twofold differences in soma capacitance between the smallest and largest motoneurons (Fig. 3) (*82*). Simulations restricted to small motoneurons may risk overestimating the uniformity of inhibition duration across the motor pool as they may not represent true neuromuscular physiology. Of note, although smaller motoneurons would be more susceptible to an incoming inhibitory input than their larger counterparts (*42*, *52*), any potential effects of motoneuron size on inhibition duration would likely be more readily detectable in our simulations.

Linear increases in common input revealed greater rates of change in firing rate among earlier recruited units with later-recruited units plateauing in their discharge rates at higher contraction levels (*86*). Furthermore, the FDI stops recruiting units at lower MVCs when compared to other muscles (*86*), suggesting that our simulations replicating an experimentally observed 10% MVC may have included enough MU diversity to detect any size-dependent effects on inhibition duration with varying firing rate.

While we cannot exclude the possibility that alternative parameters outside the explored hyperparameter space could produce different outcomes, we tested optimized parameters from two individuals with markedly different values of A (1.8 and 2.8 a.u.) and τ (7 and 19 ms). In both cases, motoneuron size had only a minor effect on inhibition duration, suggesting that the strength of inhibition is unlikely to play a role in the observed relationship.

MUs recruited at low MVCs likely reflect activity of smaller, low-threshold motoneurons, which are typically more resistant to degeneration in conditions such as motoneuron disease (*87*). This suggests that our approach—combining peripheral nerve stimulation with HDsEMG MU-level analysis—may remain viable even in symptomatic stages, offering potential insights into microcircuit-related resilience mechanisms. However, we acknowledge that the generalizability of our findings to high-threshold MUs remains limited.

Our study highlights HDsEMG as a tool that offers unparalleled resolution for participant-specific studies of spinal inhibitory microcircuits. By integrating experimental data with in silico modeling, we further extend the scope of HDsEMG in elucidating both circuit function and the biophysical properties of its neuronal elements. We hope that our work encourages broader adoption of HDsEMG as an approach to probe motor circuit function in health and disease, given that its technical advantages open frontiers in the quality and depth of data attainable from human participants.

## MATERIALS AND METHODS

### Participants

Eighteen healthy participants (10 male and 8 female, aged 25 ± 5 years, height 172 ± 10 cm) were recruited for this study. The study was approved by Imperial College London ethics committee (reference number 18IC4685), the experiments were conducted in accordance with the Declaration of Helsinki, and all the participants signed a written consent form before the experiments.

### Procedures

#### High-density sEMG

Before the application of the HDsEMG electrodes, the skin was prepared by applying abrasive paste and water. For the CSP experiment, one HDsEMG grid (64 electrodes, 13 rows, and 5 columns; 1-mm electrode diameter, 4-mm interelectrode distance; OT Bioelettronica, Italy) was placed on the FDI of the dominant hand. Two wet wristbands were used as reference and grounding electrodes on the right wrist of the participants. For the reciprocal inhibition experiment, a customized HDsEMG grid (256 electrodes, 32 rows, and 4 columns; 0.5-mm electrode diameter, 4-mm interelectrode distance; OT Bioelettronica, Italy) was placed on the TA of the dominant leg. Two HDsEMG grids (128 electrodes, 26 rows, and 5 columns; 0.5-mm electrode diameter, 4-mm interelectrode distance; OT Bioelettronica, Italy) were placed on the medial and lateral head of the soleus (SOL). Two wet wristbands were used as reference and grounding electrodes on the right ankle of the participants. All the signals from the HDsEMG electrodes were acquired at 2042.48 Hz in monopolar mode (Quattrocento, OT Bioelettronica, Italy).

#### Cutaneous silent period

To study CSP, the participants were asked to perform a pinching task between the medial side of the third phalanx of the first digit of the hand and the thumb finger while stimulation on the fifth digit of the hand was delivered. The participants were sat on a chair while conducting the experiment. A load cell (FC22, Measurement Specialties, US) was used to measure the force applied on the index finger. The force signals were acquired at 2042.48 Hz simultaneously with the HDsEMG and displayed as visual feedback. The participants were asked to perform their MVC for the pinching task. Then, stimuli were delivered using two stainless steel rings that were placed between the first and the second phalanges of the fifth digit of the hand. Stimulation pulse width was set to 200 μs, and conductive gel was applied between the ring electrodes and the skin to reduce impedance—extra care was taken to remove any conductive paste between the ring electrodes to prevent shorting. ST, defined as the minimum stimulation intensity that generated a subjective perception on the finger, was found for each participant, and stimulus intensity was set at 10 times this ST for each participant. After familiarization with the execution of the force-tracking task, the participants were asked to perform a submaximal trapezoidal isometric contraction at 10% MVC (2-s ramp up, 200-s plateau, and 2-s ramp down), while single electrical stimuli were delivered on the fifth digit of the hand (1.8 ± 0.2 s interstimulus interval; ~111 stimuli) during the plateau.

#### Reciprocal inhibition

To study reciprocal inhibition on the TA, the participants were asked to perform an isometric dorsiflexion task while electrical stimuli were delivered on the tibial nerve. The participants were sat on a chair while their leg was fixed into an ankle dynamometer (OT Bioelettronica, Italy) using straps (ankle position at 10°, 0° being the neutral foot perpendicular to the shank; knee at 75°). The dorsiflexion force signals were acquired at 2042.48 Hz simultaneously with the HDsEMG and displayed as visual feedback. The participants were asked to perform their MVC for the dorsiflexion task. A stimulation electrode (7.5 cm–by–13 cm adhesive electrode, ValueTrode, Axelgaard, Denmark) was placed as anode over the patella, while a stimulation electrode was placed as cathode (1-cm diameter customized metal ball) over the middle part of the popliteal fossa. The final stimulation position of the cathode was optimized for each participant to elicit a clear H-reflex response in the SOL (*88*). Stimulation pulse width was set to 1 ms, and conductive gel was applied to the cathode to reduce skin impedance and discomfort. The stimulation intensity was selected as 1.1× of the stimulation intensity necessary to elicit the minimum detectable H-reflex on the SOL (based on selection of monopolar channels from the medial head of the SOL). After familiarization with the performance of the force-tracking task, the participants were asked to perform a submaximal trapezoidal dorsiflexion contraction at 10% MVC (2-s ramp up, 300-s plateau, and 2-s ramp down) while single electrical stimuli were delivered to the tibial nerve (2.0 ± 0.2 s interstimulus interval; ~150 stimuli) during the plateau. This stimulus rate was appropriate to avoid exacerbating post-activation depression (*89*).

#### In silico modeling

To model CSP and reciprocal inhibition circuits in the motor pool, we implemented a Hodgkin-Huxley model with dendritic and somatic compartments (*82*). The differential equations to compute the membrane voltage for each motoneuron was computed using Eq. 1 and solved with the fourth-order Runge-Kutta method with a 0.2-ms step resolution (*90*, *91*)

$$C_{m}\;\frac{\mathrm{dv}}{\mathrm{dt}}=I_{\text{ext}}+\sum_{k}g_{k}\left(v-E_{k}\right)$$ (1)

where *C*_m_ represents the membrane capacitance, and *I*_ext_ is the external synaptic input to the cell. The conductance of a specific synaptic or intrinsic ion channel *k* is denoted by *g_k_*, while *v* represents the compartment’s voltage. The term *E_k_* corresponds to the reversal potential associated with each conductance, which may represent either the equilibrium potential of a specific ion in the case of intrinsic currents, or the effective reversal potential for excitatory and inhibitory synaptic inputs. Since each compartment has distinct ion channels and inputs, their governing equations differ. The somatic compartment includes sodium channels and both fast and slow potassium channels, while the dendritic compartment contains sodium channels along with excitatory and inhibitory synaptic inputs (Eq. 2)

$$\begin{array}{c} \frac{d{\text{Voltage}}_{\text{soma}}}{\mathrm{dt}}=\frac{1}{C_{\text{soma}}}\times -\left[g_{\text{coupling}}\cdot \left(v_{\text{soma}}-v_{\text{dendrite}}\right)+\right.\\ \;g_{\text{leak}}\cdot \left(v_{\text{soma}}-E_{\text{leak}}\right)+g_{\mathrm{Na}}\cdot m^{3}\cdot h\cdot \left(v_{\text{soma}}-E_{\mathrm{Na}}\right)+\\ \;g_{\text{Kfast}}\cdot n^{4}\cdot \left(v_{\text{soma}}-E_{\text{Kfast}}\right)+g_{\text{Kslow}}\cdot \\ \;\left.q^{2}\cdot (v_{\text{soma}}-E_{\text{Kslow}})\right]\frac{{\text{dVoltage}}_{\text{dendrite}}}{\mathrm{dt}}=\frac{1}{C_{\text{dendrite}}}\times \\ \;-\left[g_{\text{coupling}}\cdot \left(v_{\text{dendrite}}-v_{\text{soma}}\right)+g_{\text{leak}}\cdot \left(v_{\text{dendrite}}-E_{\text{leak}}\right)+\right.\\ \;g_{\mathrm{Ca}}\cdot p\cdot \left(v_{\text{dendrite}}-E_{\mathrm{Ca}}\right)+Exc\cdot G_{\text{excit}}\cdot \left(v_{\text{dendrite}}-E_{\text{excit}}\right)+\\ \;\left.Inh\cdot G_{\text{inhib}}\cdot \left(v_{\text{dendrite}}-E_{\text{inhib}}\right)\right] \end{array}$$ (2)

with *C*_soma_ and *C*_dendrite_ representing the capacitance of each compartment; *g*_coupling_ the electrical coupling between somatic and dendritic compartments—computed using axial resistivity and compartment geometry, modeling the soma and dendrite as cylinders; *v*_soma_ and *v*_dendrite_ are the voltages at a given time *t* for each motoneuron and compartment; *g*_leak_ are the conductances for leak currents; *g*_Na_ represents voltage-gated Na^+^ channels; *g*_Kfast_ and *g*_Kslow_ indicate fast and slow-kinetics voltage-gated K^+^ channels, respectively.

Gating variables followed first-order kinetic equations, representing the probability of ion channels being open or closed; *m* and *h* govern the activation and inactivation of sodium channels, *n* and *q* control the activation of fast and slow potassium channels, respectively, and *p* regulates calcium channel activation; *E*_Na_, *E*_K_, *E*_Ca_, and *E*_leak_ are the equilibrium potentials for Na^+^, K^+^, Ca^+^, and leak channels; *E*_excit_ and *E*_inhib_ are the reversal potentials for excitatory and inhibitory inputs; *Exc* represents the excitatory common input (a.u.) given to the motoneuron pool—in this case, a trapezoid signal—and *Inh* (a.u.) is the inhibitory input received reproducing the CSP or reciprocal inhibition; and *G*_excit_ and *G*_inhib_ are the synaptic conductance for excitation and inhibition. To introduce biological variability, each motoneuron receives an excitatory input and a white Gaussian independent noise that follows a distribution of N (0,0.25σ^2^). Motoneuron firing occurred when somatic voltage exceeds a threshold calculated as the product of the minimum current to elicit an action potential and input resistance.

The motoneuron parameters were obtained from in vivo motoneuron recordings obtained from S-type motoneurons from decerebrated cats (*44*–*49*) and linearly interpolated across *n* simulated motoneurons, ranging from the smallest to the largest motoneuron sizes (see table S6). In particular, soma and dendrite length and diameter were linearly varied within the range of parameters for the *n* simulated motoneurons, using these values to estimate the soma and dendrite conductance and capacitance for each neuron. S-type motoneurons were specifically chosen as they best represent the motoneuron pool recruited at low contraction intensities (10% MVC) during experimental HDsEMG recordings.

A motor pool of 20 motoneurons was simulated, each receiving trapezoidal common synaptic input mimicking the force trajectory performed in the experimental conditions: a 2-s ramp-up and ramp-down phase with a 200-s plateau phase in between for the CSP modeling, and 300-s plateau for the reciprocal inhibition modeling. Before the modeling of the inhibitory responses, we adjusted the amplitude of the excitatory common input (between 0.15 and 0.3 a.u.) to replicate the firing rates measured with HDsEMG from the FDI and TA muscles. We compared the experimental firing rate—averaged across all participants—and checked that it closely mimicked the simulated data by computing the error between them (error ~ 0) and also by implementing a Kolmogorov-Smirnov test. This test confirmed that both datasets followed the same distribution (*P* = 0.48). After testing different amplitudes for the excitatory common input, we found that an amplitude of 0.2 a.u. replicated the experimental average firing rate well for both muscles. In addition, for reciprocal inhibition, an amplitude of 0.15 a.u. for the common input was simulated to minimize noise effect of background firing rate on estimating inhibition duration, without deviating from experimentally observed firing rates (fig. S5).

The inhibitory input (*Inh*) replicating the inhibitory responses elicited through electrical stimulation was modeled as an alpha function (a.u.) with time constant (τ) and amplitude (A). This approach was chosen to capture the rapid onset of inhibition followed by a gradual decay (see Figs. 2A and 5A)

$$\mathrm{Inh}\left(t\right)=A\left(\frac{t}{\tau }\right)\cdot e^{-\frac{t}{\tau }}$$ (3)

To reproduce the inhibitory responses observed in the experimental CSP and reciprocal inhibition, we conducted a hyperparameter sweep to explore different combinations of inhibition amplitude (A) and time constant (τ). This allowed us to map a parameter space that could generate motoneuron inhibition profiles consistent with the experimental data. The inhibitory input (*Inh*) to the simulated motoneurons was delivered every 1.8 s for CSP (Fig. 2A) and every 2 s for reciprocal inhibition (Fig. 5A). Inhibition amplitudes (A) ranged from 1 to 3 a.u., varying in 0.2-a.u. increments, while the time constant (τ) varied between 7 and 20 ms for CSP and 1 to 4 ms for reciprocal inhibition in 1-ms steps. The resulting motoneuron spike trains were downsampled from 20K to 2 kHz to match the experimental MU spike trains, and the inhibitory responses were analyzed manually.

To investigate the impact of motoneuron biophysical properties, namely motoneuron size, on the duration of inhibition for CSP, we simulated a larger pool of 40 motoneurons using the previously described paradigm. We doubled the number of motoneurons for these simulations to achieve a higher resolution in mapping the intrinsic property gradients of motoneurons. In this case, the inhibitory input strength was held constant within each simulated motoneuron pool, triggered every 1.8 s with A and τ values were selected so they closely matched experimental data from one of the participants (participant 4, randomly selected; Fig. 3A). Meanwhile, for each motoneuron pool simulated, the common excitatory synaptic input was varied (*Exc* varying from 0.21 to 0.28 a.u., with 0.01-a.u. increments). The output motoneuron spike trains were downsampled to 2 kHz, and the inhibitory responses were analyzed manually following the methods described below. The goal of these simulations was to generate motoneurons with different sizes that, despite receiving the same inhibitory drive, discharged at similar firing rates due to differences in the strength of the common excitatory input.

### Data analysis

#### HDsEMG decomposition

Monopolar HDsEMG signals were decomposed into individual MU spiking activity using a validated blind source separation algorithm (*92*–*94*). This algorithm provides a solution to the inverse EMG convolutive mixing model by estimating the separation vectors (MU filters) that yield the sources of the EMG signals (individual MU spike trains). Before the application of the decomposition algorithm, signals were band-pass filtered (20 to 500 Hz, second-order Butterworth) and visually inspected to reject channels with low signal-to-noise ratio. Since the stimulation artifact and the presence of evoked compound action potentials could bias the identification of MUs, the signals contained in the interval 5 to 100 ms relative to the stimuli where not inputted into the decomposition algorithm. Because of the extended length of the recordings, a window of 30 s selected from the plateau of the isometric contraction was selected to extract the MU filters. Then, the MU filters were extended to the whole recording, and the MUs identified in the 30-s plateau were tracked throughout the entire trial. All HDsEMG signals were decomposed using the same procedure.

Among all the MUs detected, only those with pulse-to-noise ratio ≥ 28 dB (*95*), coefficient of variation of their discharge rate < 0.3 and stable discharge patterns were retained for analysis. Although isometric contractions tend to yield stable MU firing rates, stimulation-evoked responses can alter this pattern via inhibition or excitation. Manual editing, which is a common postprocessing step in which MU spike trains identified by the algorithm are reviewed and possibly corrected, was minimized in this study to avoid biasing the characterization of these evoked effects. We implemented a highly objective and semiautomated editing approach to ensure consistency: MUs with implausible firing statistics (e.g., abnormally low or high rates suggestive of decomposition errors) were systematically excluded on the basis of preset criteria. No manual corrections were made to the spike trains themselves. The only manual step involved was the visual marking of inhibition onset (from PSTH-CUSUM) and termination (from PSF-CUSUM) for each MU. MU spike trains and HDsEMG signals were downsampled to 2 kHz for the subsequent analysis.

To estimate the onset of the MU action potential (MUAP) relative to the discharge times identified by the decomposition algorithm, MUAPs were calculated by spike-averaging all the HDsEMG channels, and the channel with the highest peak-to-peak amplitude was selected to estimate the MUAP onset time (*96*). Using this channel, a threshold set at 20% of the maximum amplitude of the MUAP was set to define the onset time of the MUAP, which was checked visually for all the MUs included in the analysis.

Throughout the FDI and TA contractions, bipolar sEMG was continuously monitored to detect potential signs of fatigue. Specifically, we compared changes in sEMG amplitude, as well as individual MU firing rates, at the start and end of the voluntary contraction. While no clear increases in sEMG amplitude were detected, we observed mild reductions in MU firing rate (~10%; fig. S6), as expected for sustained low-intensity contractions, there were no abrupt alterations that would indicate the development of substantial neuromuscular fatigue. Furthermore, we are likely recording from homogenous motor pools (likely S-type MUs recruited at low MVCs), and any impacts of fatigue would affect MUs in a uniform manner. Therefore, in line with previous studies using longer sustained contractions at low MVCs (*13*, *14*, *20*), the inhibitory measures were not largely affected by fatigue-related changes.

#### Inhibitory responses

An automatic procedure was applied to remove outliers in the discharge pattern by removing instantaneous discharges above the median discharge rate plus 2.5× the SD and below 50% of the median discharge rate. The resultant MU spike trains were used to estimate the inhibitory responses with the PSTH used to estimate the latency (i.e., start of inhibition) and the PSF for duration (i.e., end of inhibition) as previously described (*6*, *7*, *20*). In summary, the PSTH, representing the number of MU firing occurrences locked around the stimulation, and the PSF, representing the instantaneous discharge rates, were computed in bin widths of 1 ms. The CUSUM of both PSTH and PSF were calculated. The reasoning for using both PSTH and PSF is the following: PSTH reflects the probability of MU firing relative to the stimulus, thus making it suitable for determining inhibition onset. However, it cannot capture the full duration of inhibition since it relays information from changes in firing probability. PSF tracks instantaneous discharge rate throughout time and since inhibition affects the firing rate rather than completely silencing all the units especially toward the rise phase of the IPSP (i.e., late, weaker phase), it provides a continuous measure of how long the synaptic inhibition persists. Therefore, by using PSTH for onset and PSF for end of inhibition, we ensure a more precise characterization of the inhibition duration (*6*, *7*, *20*). Since the stimulation artifact and the presence of direct motor response (M-wave) could be merged in the MU spike train leading to inaccurate discharge identification, the CUSUM was not updated in the window comprising ±20 ms around the stimulation. Inhibition events were identified manually by visual inspection and set as genuine inhibitory responses when the amplitude between two inflexion points in the PSF-CUSUM and PSTH-CUSUM was greater than the maximum variation in the prestimulus window (−200 and −30 ms, limits fig. S1). This conservative threshold ensured that only clear and measurable inhibitory responses were analyzed (*26*). Genuine inhibitory events were detected according to the criteria above in ~80 and ~70% of all MUs sampled for CSP and reciprocal inhibition, respectively. The motoneurons that have a weak inhibition but not significant (i.e., inhibition size smaller than the CUSUM limits) were not included into linear model generation, thus resulting in variable number of motoneurons in each realization for modeling. These excluded units did not exhibit systematic differences in baseline discharge characteristics compared to the included MUs. The inhibition latency and end time points were selected manually in the PSTH-CUSUM and PSF-CUSUM as the first clear deflection point of a trough, and the last deflection point or peak of the trough, respectively. The inhibition duration was computed as the time interval between the inhibition latency estimated in the PSTH and the inhibition end point selected in the PSF. The inhibition amplitude was computed as the difference between the CUSUM values at the end and onset of the inhibition time points for both PSTH and PSF and reported in the Supplemental Materials (tables S7 to S10).

To estimate the inhibitory responses in the global EMG, raw sEMG signals were digitally band-pass filtered (20 to 500 Hz, second-order Butterworth) and then rectified and segmented in 600-ms windows around the stimuli. The rectified sEMG signals were relativized to the baseline activity, which was calculated as the averaged rectified sEMG in the time interval from 200 to 25 ms before the stimulus. The start of inhibition was determined manually as the time when the signal was lower than baseline for at least 5 ms, while the end of the inhibition was manually set as the time when the signal was no longer lower than baseline for at least 5 ms. Inhibition duration was estimated as the time difference between the start and the end points (see fig. S1).

Since inhibition measured through HDsEMG during voluntary contractions is influenced by discharge rate, parameters were interpreted in relation to MU firing frequencies. The experimental inhibitory responses (firing rate and inhibition duration) were fitted to a linear model (LM_exp_), and to evaluate how discharge rate explained variations in inhibition duration, we relied on the *R*^2^ metric as a measure of goodness of fit (*31*, *32*). This *R*^2^ was calculated as

$$R^{2}=1-\frac{\sum_{i}{\left(y_{\text{exp},i}-{\hat{y}}_{\text{pred},i}\right)}^{2}}{\sum_{i}{\left(y_{\text{exp},i}-{\bar{y}}_{\text{exp},i}\right)}^{2}}$$

where *y*_exp,*i*_ and ${\bar{y}}_{\text{exp},i}$ are the experimental data point and experimental mean for the *i*th observation, respectively, and ${\hat{y}}_{\text{pred}}$ is the predicted inhibition duration from the linear fit (fig. S2A). Although the relationship between inhibition duration and MU firing rate is the ideal proxy for estimating inhibition, we still report in the Supplementary Materials pertaining to PSTH and PSF amplitudes (tables S7 to S10).

For all in silico simulations, the inhibition response for each motoneuron was manually quantified using PSTH and PSF methods, following the same procedure as for the experimental data analysis described above (Fig. 2B). Then, the inhibitory responses (firing rate and inhibition duration) obtained from each realization of the simulation were fitted to a linear model (LM_sim_; figs. S3A and S5, A and B). To select the optimal LM_sim_ for each participant, we first inputted their experimental firing rate into all the candidate LM_sim_ obtained from all the realizations of the biophysical model across the different combinations of parameters A and τ. The selection process prioritized minimizing the MSE, which has the same numerator as the *R*^2^, ensuring that the chosen LM_sim_ provided the best prediction of inhibition duration across participants by reducing overall deviation from experimental data while preserving the underlying relationship between firing rate and inhibition duration (see fig. S2D)

$$\mathrm{MSE}=\frac{\sum_{i}{\left(y_{\text{exp},i}-{\hat{y}}_{\text{pred},i}\right)}^{2}}{N}$$

Inhibition duration, PSTH and PSF amplitudes for CSP, and reciprocal inhibition for the simulated data pertaining to the LM_sim_ selected for each participant are reported in the Supplementary Materials (tables S11 to S16).

Although MUs with higher discharge rates are generally assumed to be smaller and recruited earlier (i.e., following the onion-skin model), this relationship may not universally apply to all MUs in all conditions (*97*). To assess this relationship in our data, we estimated MU recruitment thresholds from the brief (2 s) ramp-up phase to 10% MVC before each sustained contraction. Although this ramp phase was not emphasized during data collection, we were able to reliably extract recruitment thresholds for the FDI of four participants and for the TA of seven participants. These estimates were then compared to the MU discharge rates measured during the steady contraction plateau, revealing a negative correlation between MU firing rate and recruitment threshold (fig. S7, A and B, and tables S17 to S18), indicating that the earliest recruited MUs do fire at higher rates.

For the in silico simulations exploring the impact of motoneuron biophysical properties on inhibition duration, the resulting motoneurons across all the simulations were clustered into three main groups based on their firing rate (11 to 12, 13 to 14, and 14 to 15 Hz). This clustering permitted to evaluate how cell size (i.e., capacitance) could explain variability in inhibition duration within fixed MU discharge rate intervals (see Fig. 3B).

In our study, we used the PSTH-PSF method using ~111 stimuli for the FDI and ~150 stimuli for the TA, which resulted in 1-ms PSTH bins typically containing one to two MU firings near the inhibition phase (see Figs. 1B and 4B, top). To determine whether bin width or number of stimuli influenced the estimation of functional inhibition, we conducted additional simulations. Specifically, we simulated a pool of 40 motoneurons, each receiving a trapezoidal common excitatory input and an inhibitory input delivered periodically during the steady-state phase (same as in Fig. 2A), and we compared inhibition durations using bin widths of 1 and 2 ms, and extended the number of stimuli delivered—250 and 500—to populate the bins with more MU firings. As shown in fig. S7 (C to D), we found that neither bin width nor stimulus count had a substantial effect on the estimation of inhibition duration. Moreover, in the context of HDsEMG recordings, increasing the number of stimuli would have substantially extended recording times, potentially enhancing muscle fatigue (see fig. S6) and causing discomfort, which could in turn could affect the quality or reliability of our measurements.

### Statistical analysis

Statistical analyses, computational simulations, plots, and figures were generated using MATLAB R2022b (Mathworks, USA) and Microsoft Excel version 2208 (Microsoft, USA). The main statistics focused on relationships between MU activity variables, including

1) Inhibition duration and firing rate;

2) PSTH inhibition amplitude and firing rate;

3) PSF inhibition amplitude and firing rate;

4) Motoneuron capacitance and inhibition duration;

5) Firing rate and recruitment threshold.

For most of the relationships, we performed two different complementary analyses as follows:

#### Grouped analysis

Here, we considered all the MUs as independent, and pooled experimental MU data from all participants (Figs. 1D and 4D) or simulated MUs pertaining to the optimized LM_sim_ for each participant (Figs. 2C and 5C) and fitted a linear regression model (LM) using the fitlm function in MATLAB (*98*). The *R*^2^ was used to infer the strength of the linear regression in explaining the variance in each relationship and therefore the LM’s ability to capture meaningful trends within the pooled data (*99*). For better interpretation of *R*^2^ values obtained, we will consider the threshold values of 0.67, 0.33, or 0.19 as strong, moderate, and weak coefficients (fig. S2) (*33*, *34*). In addition, to assess the statistical significance of the linear fit, we report *P* values obtained from fitlm (*98*). These *P* values are computed from the *t*-statistic (*t*), which evaluates whether the slope (β) of the regression line significantly differs from zero (*100*)

$$t=\frac{\beta }{\mathrm{SE}\left(\beta \right)}$$

where *SE* is the standard error of the slope. The corresponding *P* value is derived from a *t*-distribution with degrees of freedom (*n* − 2), with statistical significance set at 0.05 (*100*).

#### Participant-specific analysis

We analyzed the data by taking into consideration intergroup variabilities. Here, we fitted a linear regression model for the experimental data pertaining to each participant (LM_exp_; Figs. 1E and 4E), or to the simulated MUs for each realization of the in silico biophysical model (figs. S3 and S5), generating linear regression models for the simulated data (LM_sim_). Only realizations with more than two MUs with detectable inhibition were fitted. For the experimental data (Figs. 1E and 4E) and the simulated MU data pertaining to the optimized LM_sim_ selected for each participant (tables S11 to S16), we also fitted a random intercept (model 1) and a random intercept and random slope (model 2) LMM using the fitlme function from MATLAB under the restricted maximum likelihood fitting method (*101*) as follows

Model 1: $Y_{i,k}=\beta_{0}+{\text{Predictor}}_{i,k}\times \beta_{1}+\gamma_{\text{subject},k}+\varepsilon_{i,k}$

Model 2: $Y_{i,k}=\beta_{0}+{\text{Predictor}}_{i,k}\times \beta_{1}+\gamma_{\text{subject},k}+\gamma_{\text{subject},k}^{\text{Predictor}}\times {\text{Predictor}}_{i,k}+\varepsilon_{i,k}$

with *Y*_*i*,*k*_ representing the collected data point for “inhibition duration,” “PSTH inhibition amplitude,” or “PSF inhibition amplitude,” belonging to the ith observation obtained from the kth subject; β_0_ is the fixed intercept; *Predictor*_*i*,*k*_ is the predictor variable (“firing rate”) for observation *i* within *k* and β_1_ is its corresponding coefficient; γ_subject,*k*_ represents the random effect intercept for the kth subject to capture participant-specific deviations from the fixed intercept; $\gamma_{\text{subject},k}^{\text{Predictor}}$ is the random effect slope for the predictor variable in the *k*th subject to account for participant-specific variability in the predictor and outcome relationships (e.g., slope for inhibition duration versus firing rate is adjusted for each participant according to their firing rate); and ε_*i*,*k*_ is the residual error for observation *i* in subject *k*.

From the fitlme output, we reported the predicted value for the intercept and the estimated difference for the fixed effects along with respective 95% CI. The fitlme also provides the SE and its 95% CI for each of the random effects, which we used to estimate the ICC. The ICC quantifies the proportion of total variance attributable to between-participants variability, thus relaying information into the sources of variability in our hierarchical datasets (*68*, *102*). For model 2, we also report the correlation and 95% CI for the random slope and intercept interaction, noting that overfitting sometimes led to singular fits (“Singular”; see tables), indicated by a correlation of ±1. We also report the *R*^2^, a metric used to gauge the variance explained by the LMM (*103*).

For graphical representation and data interpretation, we selected either LMM model 1 or model 2 based on an LMM comparison. We used the compare function in MATLAB (*104*) to perform a likelihood ratio test, which compares each of the LMM’s log-likelihood, Akaike information criterion (AIC), and Bayesian information criterion (BIC) and provides a *P* value of the test. These metrics evaluate the balance between model fit and complexity (*105*), with the LMM exhibiting the lowest AIC/BIC, highest log-likelihood, and a *P* value lower than 0.05 from the likelihood ratio test statistic being selected.

To select the optimized LM_sim_ for each participant, we compared the *R*^2^ experimentally determined with recorded data (LM_exp_; Figs. 1E and 4E) with the *R*^2^ of the all the generated LMs_sim_ predicted with experimental firing rates (figs. S2 to S5 and Figs. 2D and 5D). The *R*^2^ metric is a common goodness-of-fit validation metric for biophysical models (*79*) as it quantifies the proportion of variance in experimental data captured by the simulations, thus allowing to assess how well our in silico models aligned with the observed experimental trends for each participant (*79*, *106*). For the simulations with varying motoneuron size (Fig. 3 and fig. S4), data obtained were grouped into three different firing frequency groups (11 to 12, 12 to 13, and 13 to 14 Hz) with varying motoneuron (MN) capacitance. A random intercept LMM was used to compare the changes in inhibition duration

$$Y_{i,k}=\beta_{0}+{\text{Firing rate}}_{i,k}\times \beta_{1}+\gamma_{\mathrm{MN}\;\text{capacitance},k}+\varepsilon_{i,k}$$

with *Y*_*i*,*k*_ representing inhibition duration for the ith observation obtained for the kth motoneuron capacitance value; β_0_ is the fixed intercept; *Firing rate*_*i*,*k*_ is the firing rate group belonging to the observation *i* within *k* with β_1_ being its coefficient; γ_MN capacitance,*k*_ represents the random effect term for the kth motoneuron capacitance value; and ε_*i*,*k*_ is the residual error for observation *i* in motoneuron group *k*. In addition, to further understand the variability introduce by motoneuron size in predicting inhibition duration, we calculated the ICC for each firing rate group through a one-way random effects ICC model (1, 1) (*102*, *107*), which was estimated through the mean squares (MS) obtained from an one-way analysis of variance (ANOVA) performed with the anova function in MATLAB (*108*)

$$\mathrm{ICC}\left(1,1\right)=\frac{{\mathrm{MS}}_{\text{between}\;\mathrm{MN}\;\text{groups}}-{\mathrm{MS}}_{\text{within}\;\mathrm{MN}\;\text{groups}}\;}{{\mathrm{MS}}_{\text{between}\;\mathrm{MN}\;\text{groups}}+\left(k+1\right)\times {\mathrm{MS}}_{\text{within}\;\mathrm{MN}\;\text{groups}}}$$

To provide a comparative metric when illustrating the optimized LM_sim_ and LM_exp_ for CSP and reciprocal inhibition (Figs. 2D and 5D), we used Hedges’ *g* to compare the distributions of squared residuals (SR) obtained from experimental data points against those from linear regressions, as follows

Hedges'g(SRLMSIM - SRLMEXP)=μSRLMSIM−μSRLMEXP(nSRLMSIM−1)σSRLMSIM2+(nSRLMEXP−1)σSRLMEXP2(nSRLMSIM−1)+(nSRLMEXP−1)

Hedges’ *g* can be used to calculate the effect size of the difference in residuals, with guidelines referring to small, medium, and large effects as 0.20, 0.50, and 0.80, respectively (*109*).

Data plots comparing inhibition duration estimated with the rectified EMG and PSF methods (Fig. 1C), sEMG root mean square and MU firing rate at the start and end of the voluntary contraction (fig. S6), and functional inhibition durations with different bin width and stimuli (fig. S7, C and D) are shown as estimations plots. These plots display the minimum, first quartile, median, third quartile, and maximum value, with data points color-coded by participant. In addition, bootstrapped (10,000 replicas, paired or unpaired) mean difference and/or Hedges’ *g* distributions (Kernell smooth) are shown, along with the mean (dot) and 95% CI (whiskers). The “0” value is aligned with the mean of the control group, while the predicted mean difference is indicated by dotted horizontal lines. Throughout the paper, the values for inhibition duration pertaining to these estimation plots are reported as means ± SD. The PSTH-CUSUM plots depict MU firing occurrence in bin widths of 1 ms plotted against time with respective overlapped CUSUM trace, whereas the PSF-CUSUM plots illustrate individual MU firing events throughout time with respective CUSUM line. Correlation graphics are shown as scatter plots with all individual data points plus linear model and/or LMM regression lines. Inset plots depict distributions of squared residuals as kernel smooth density plots with Hedges’ *g* values positioned above. Hyperparameter heatmaps were generated to display linear fit parameters across the different A and τ combinations for each realization of the in silico simulations.
