# Supplementary material for: Personalized mapping of inhibitory spinal cord circuits in humans via noninvasive neural decoding and in silico modeling
Source: Sci Adv. 2025 Sep 19;11(38):eadz5524. doi: 10.1126/sciadv.adz5524 (PMC13155565; doi:10.1126/sciadv.adz5524)
Supplement: Supplementary file 1 — Figs. S1 to S7 Tables S1 to S18 [file sciadv.adz5524_sm.pdf]

Science Advances
AAAS

Supplementary Materials for

Personalized mapping of inhibitory spinal cord circuits in humans via noninvasive neural decoding and in silico modeling

Alejandro Pascual-Valdunciel *et al.*

Corresponding author: Alejandro Pascual-Valdunciel, a.pascual-valdunciel@imperial.ac.uk;
Dario Farina, d.farina@imperial.ac.uk; Filipe Nascimento, f.nascimento@ucl.ac.uk;
M. Görkem Özyurt, g.ozyurt@ucl.ac.uk

Sci. Adv. **11**, eadz5524 (2025)
DOI: 10.1126/sciadv.adz5524

This PDF file includes:

Figs. S1 to S7
Tables S1 to S18

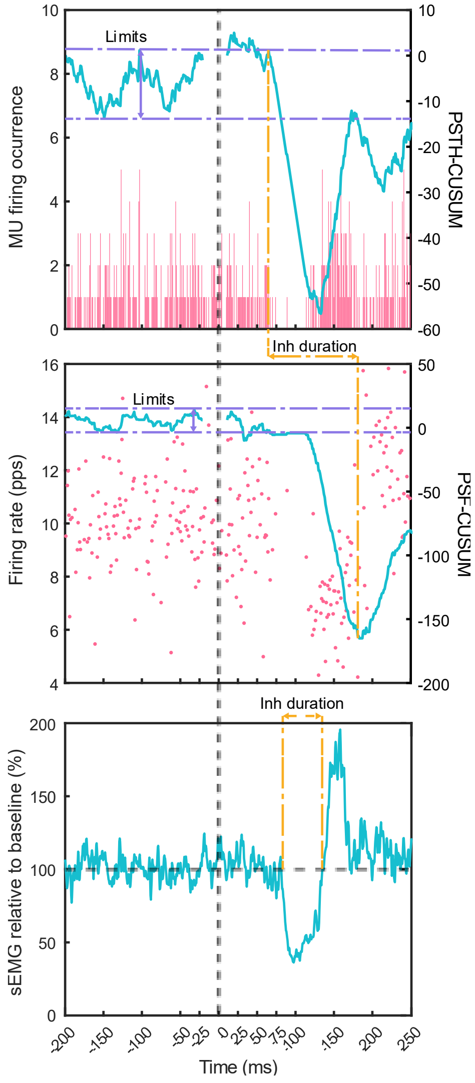

Figure S1 – Motor unit firings: PSTH and PSF with CUSUM analysis. Example of CSP motor unit firings with PSTH (top) and PSF (middle) and respective CUSUM, showing the defined start and end of inhibition (dotted yellow lines) and CUSUM limits (dotted purple line). Bottom trace displays the sEMG obtained from the same recording and the estimate of inhibition duration. Black vertical dotted line denotes onset of nerve stimulation.

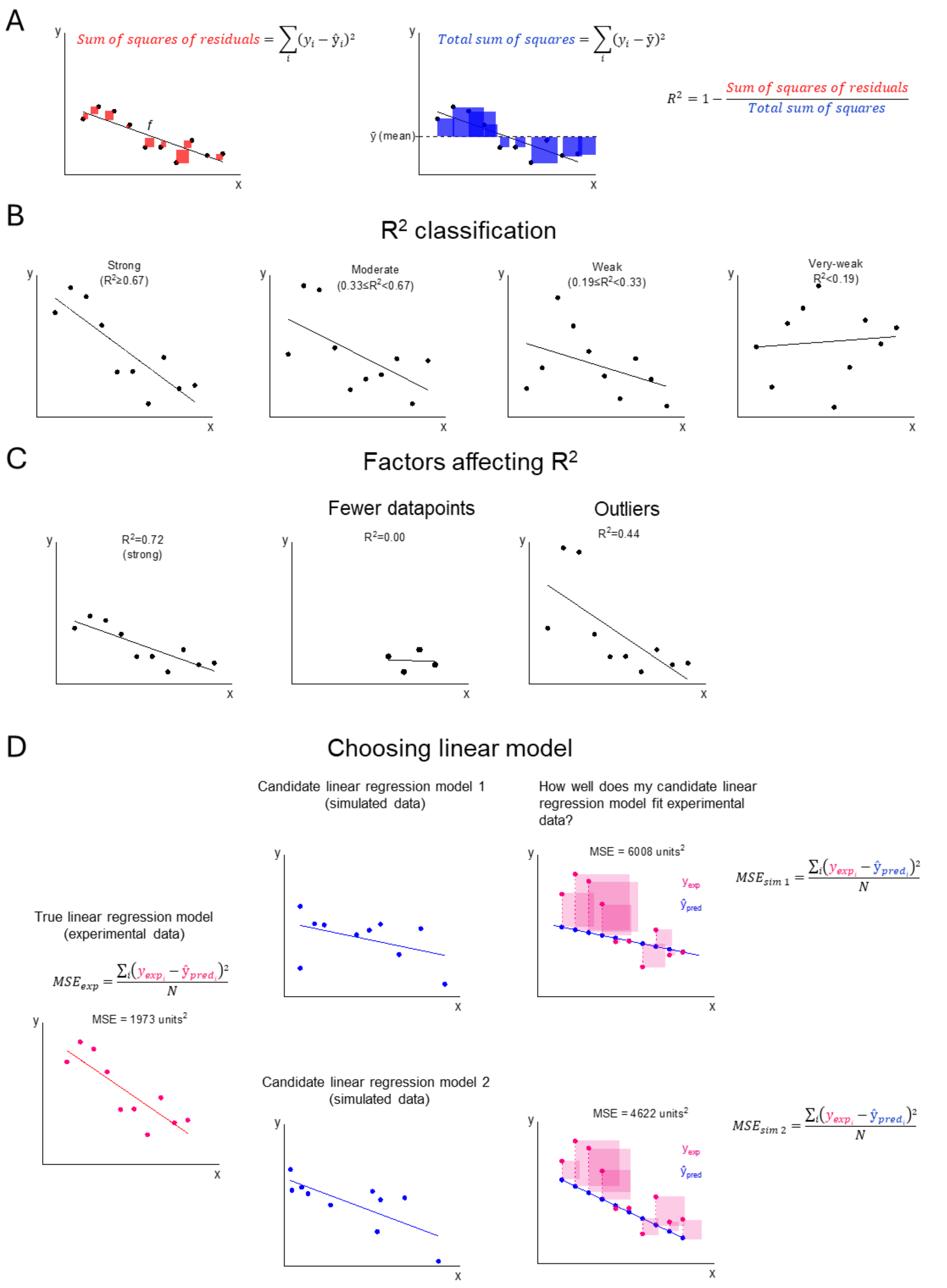

Figure S2 – Statistical assessment of variability and model selection in linear regression. (A) example of datapoints and respective linear fit (*f*), illustrating the sum of squares of residuals (red, left) and total sum of squares (blue, middle) used to estimate the coefficient of determination (R^2; right). **(B)** examples of R^2 of different strengths: strong,

moderate, weak and very weak R^2; **(C)** Illustration of factors influencing the R^2 such as sample size and datapoint outliers; **(D)** Steps for selecting the best linear model from simulated data: 1) experimental data is used to establish a reference linear model (pink, left), where the mean squared error (MSE) quantifies the true relationship between the predictor (x) and the dependent variable (y); 2) simulated datasets generate multiple candidate regression models (blue), each with its own independent and dependent variables; 3) the linear fit from each simulated model is evaluated against the experimentally measured predictor, with the best linear model selected by prioritizing the one that minimizes the MSE.

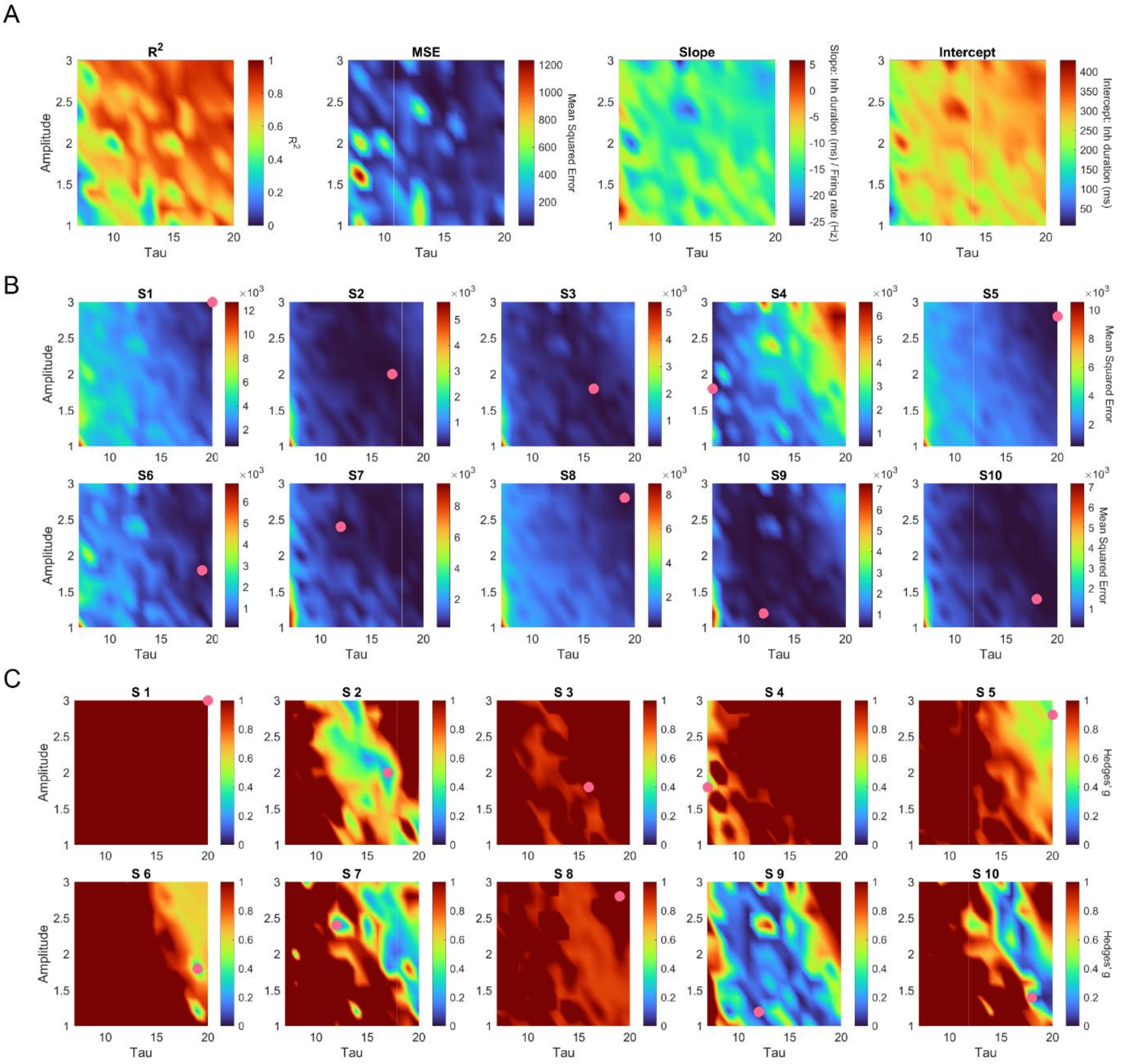

Figure S3 – Heatmap and optimization analysis of R^2 for CSP linear models from an *in silico* biophysical model. (A) Heatmaps depicting the R^2 (left), Mean Squared Error (MSE, middle left), slope (middle right) and intercept (right) of linear regressions for all combinations of amplitude (1–3 a.u.) and tau (τ, 7–20ms) across 154 realizations, with colour-coded intensity (blue to red). **(B, C)** Hyperparameter optimization plots for each of the 10 subjects, illustrating **(B)** Mean Squared Error values and **(C)** Hedges' *g* for the square of residuals comparisons between LM_{exp} and linear fits obtains obtained across amplitude and τ variations, highlighting the best-fit LM_{sim} selected for validation against CSP HDsEMG data (pink dot).

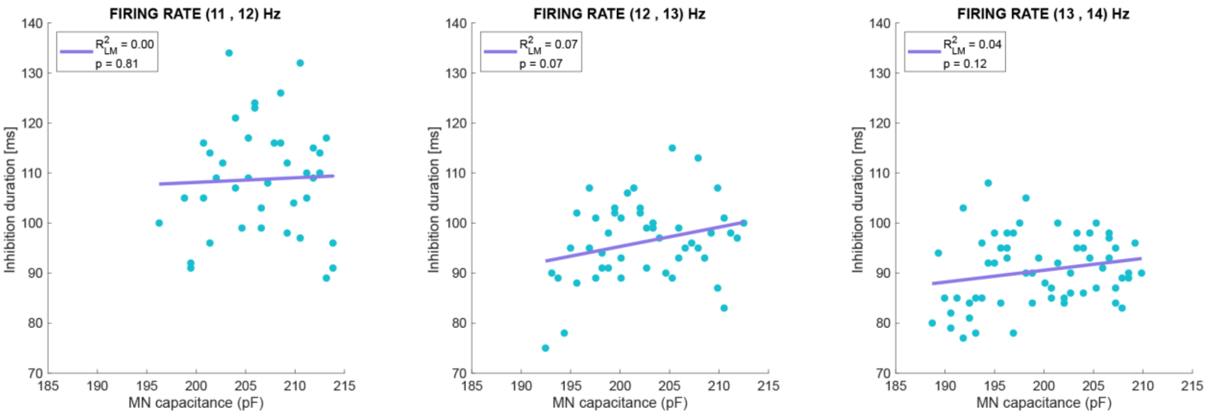

Figure S4 – Influence of motoneuron size on inhibition duration *in silico* (optimized parameters for subject 4). Inhibition duration across simulated motoneurons with varying size (blue dots) with each plot representing a different firing rate, with purple line representing the linear regression between capacitance and inhibition duration. Parameters include an inhibitory input amplitude of 1.8a.u. and duration of 7ms.

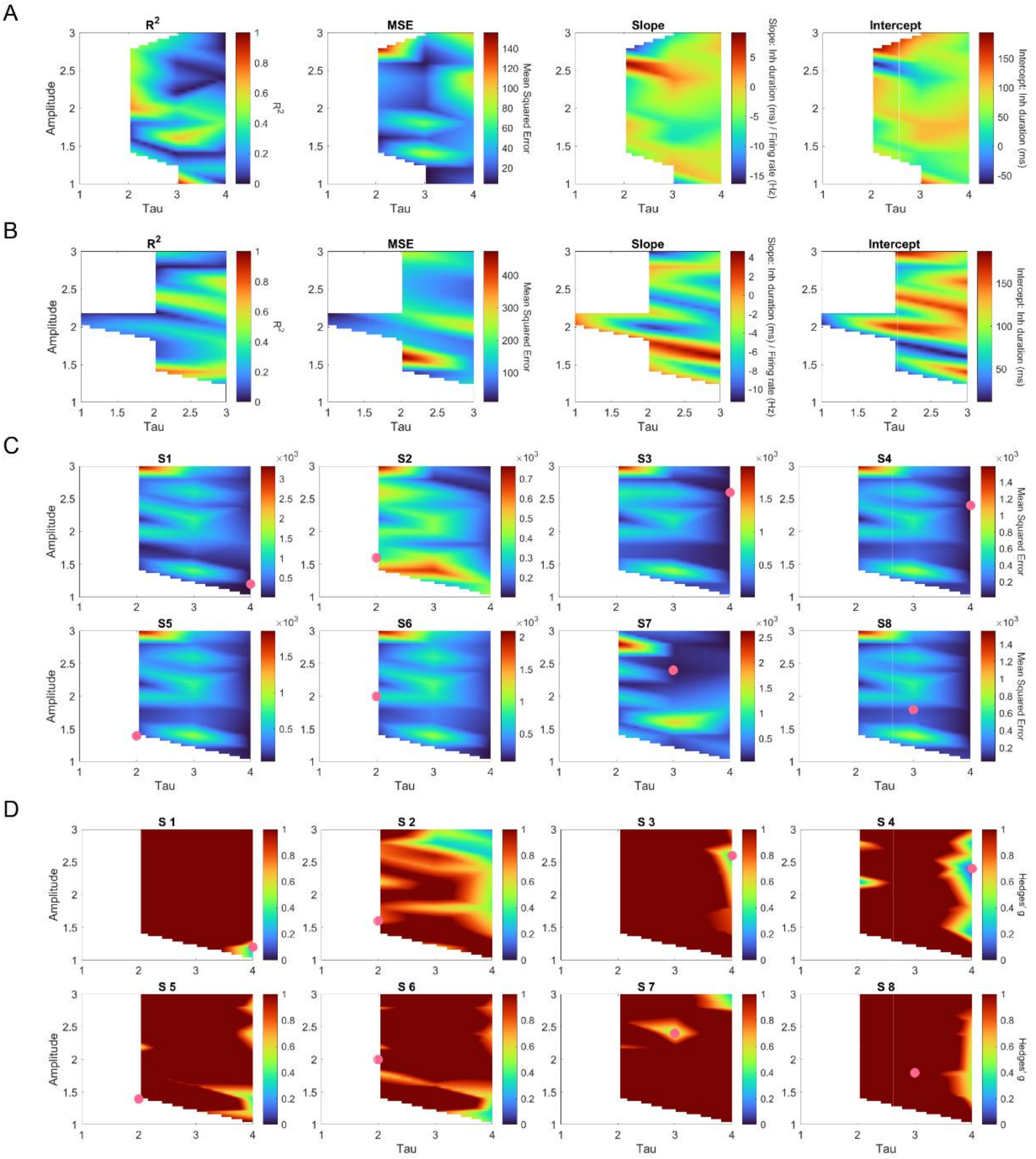

Figure S5 – Heatmap and optimization analysis of R^2 for reciprocal inhibition linear fits obtained through *in silico* biophysical modelling. (A-B) Heatmaps depicting the R^2 (left), Mean Squared Error (MSE, middle left), slope (middle right) and intercept (right) of linear regressions for all combinations of amplitude (1–3a.u.) and tau (τ, 1–4ms) across 39 realizations with excitatory common input of **(A)** 0.15a.u. and across 33 realizations with **(B)** excitatory common 0.2a.u., with colour-coded intensity (blue to red). **(C, D)** Hyperparameter optimization plots for each of the 8 subjects, illustrating **(C)** Mean Squared Error values and **(D)** Hedges' *g* for the square of residuals comparisons between LM_{exp} and linear fits obtains obtained across amplitude and τ variations, highlighting the best-fit LM_{sim} selected for validation against reciprocal inhibition HDsEMG data (pink dot). All *in silico* models except for S2 and S7 received 0.15a.u. of excitatory common input.

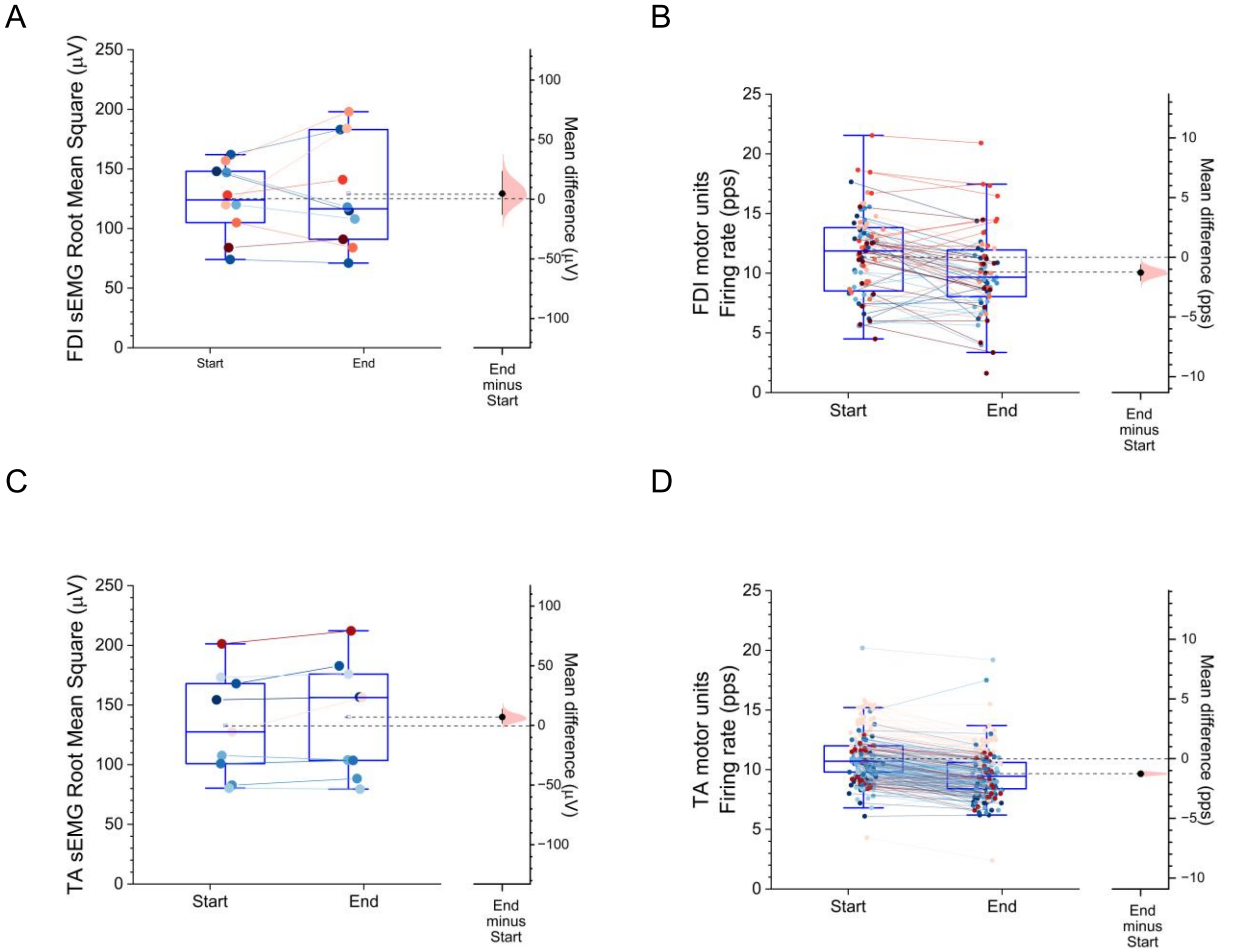

Figure S6 – Alterations in global surface EMG root mean square and individual MU firing rate at the start and end of voluntary contractions. Estimation plots showing **(A)** surface EMG root mean square (5μV [-13, 24] 95% CI) and **(B)** individual MU discharge rate alterations (-1.28pps [-1.95, -0.60] 95% CI) at the start and end of the plateau phase of the voluntary contractions for the FDI muscle (10 subjects, 57MUs). Data with effect sizes for **(C)** surface EMG median frequency (7μV [2, 14] 95% CI) and **(D)** individual MU discharge rate alterations (-1.27pps [-1.44, -1.09] 95% CI) at the start and end of the plateau phase of the voluntary contractions for the TA muscle (9 subjects, 196MUs). CI – confidence interval.

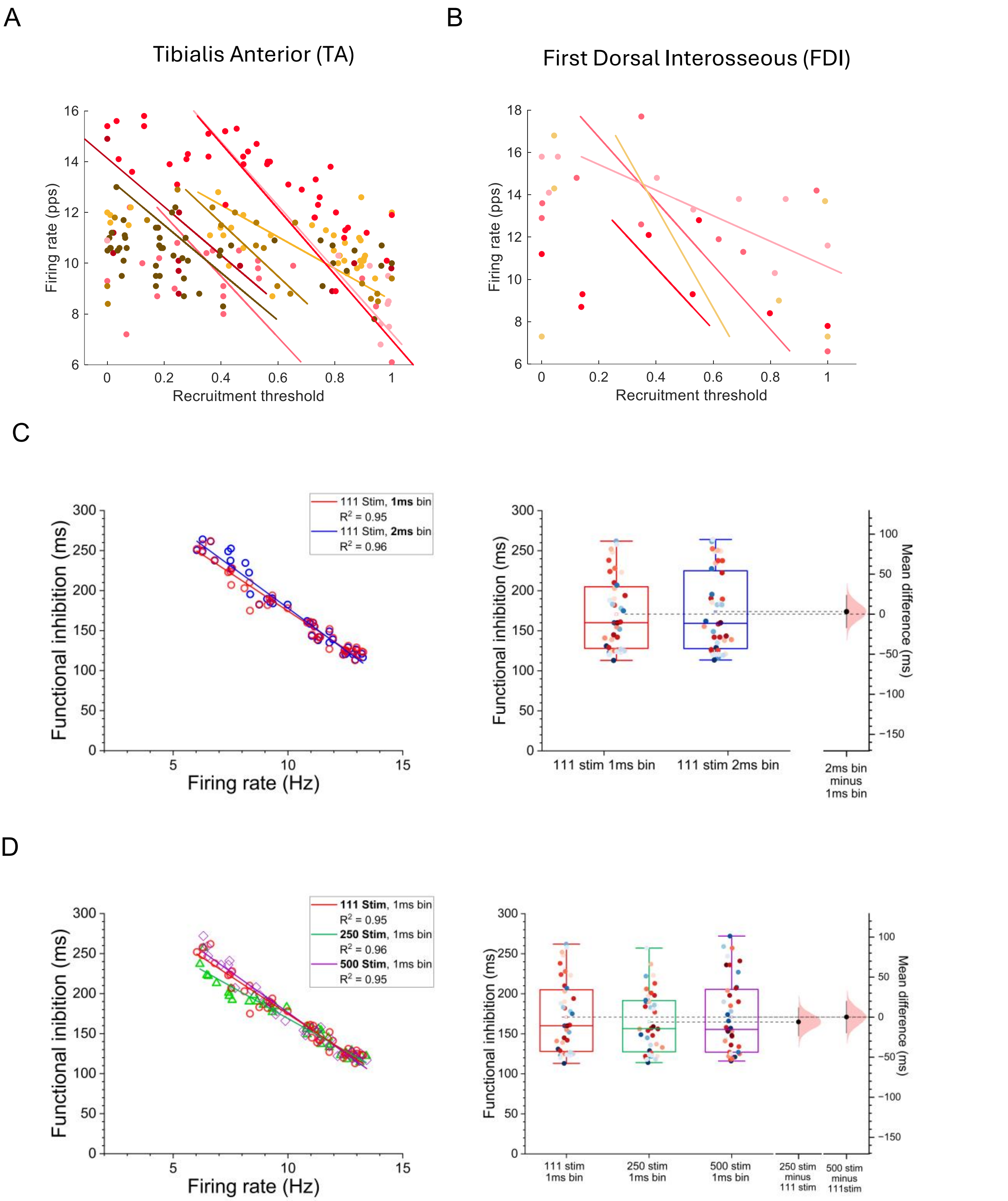

Figure S7 – Recruitment threshold and PSTH binning. Relationship between recruitment threshold (at 10% MVC) and MU discharge rate for **(A)** the TA (7 subjects) and **(B)** FDI (4 subjects) muscles; data points are colour-coded by subject. Representation of functional inhibition duration relationship with firing rate for a set of 40 simulated motoneurons (using the parameters selected for subject 8; see table S2), with respective estimation plot to compare any substantial alterations in functional inhibition, for (**C**) simulations using 111 stimuli and PSTH binning at 1ms and 2ms, and **(D)** simulations using 111, 250 and 500 stimuli and with PSTH binning fixed at 1ms.

Table S1: Fixed and random-effects variables and likelihood ratio test from random intercept (model 1) and random intercept and random slope (model 2) linear mixed models for the relationship between Inhibition duration and firing rate for cutaneous silent period (CSP) measured from first dorsal interosseous (FDI) muscle

	**Model 1:** **Inhibition Duration ~ 1 + Firing rate + (1 | Subject)**	**Model 2:** **Inhibition Duration ~ 1 + Firing rate + (Firing rate | Subject)**
**Fixed effects**		
(Intercept)	304 ms [269, 338] $p = 6.76\times10^{-27}$	304 ms [244, 364] $p = 8.29\times10^{-15}$
Firing rate	-15 ms [-17, -12] $p = 1.59\times10^{-17}$	-15 ms [-20, -10] $p = 1.26\times10^{-7}$
**Random effects**		
Subject	26 [16, 43], ICC = 0.60	80 [42, 152], ICC = 0.80
Subject x Firing Rate	--	6 [3, 12], ICC = 0.06
Correlation (Subject, Firing rate)	--	-0.96 [-1, -0.77]
Residuals	17 [14, 20], ICC = 0.40	14 [12, 17], ICC = 0.14
**Model Fit and Comparison**		
R^2	0.75	0.84
AIC	621.65	616.81
BIC	630.53	630.13
Log-Likelihood	-306.83	-302.4
p-value	--	0.012 (Model 2 preferred)

AIC: Akaike information criterion; BIC: Bayesian Information Criterion; ICC: Intraclass correlation coefficient

Table S2 - Amplitude, time constant and common input values for the LM_{sim} selected from the CSP simulations for each of the subjects, with respective R^2 from simulated MUs

Subject	Excitatory input (a.u.)	Inhibitory input amplitude (a.u.)	Inhibitory input time constant (ms)	R^2	p-value
1	0.2	3	20	0.83	1.493E-05
2	0.2	2	17	0.89	6.99E-09
3	0.2	1.8	16	0.70	1.89 E-04
4	0.2	1.8	7	0.37	0.0269
5	0.2	2.8	20	0.78	6.26E-05
6	0.2	1.8	19	0.60	1.77 E-03
7	0.2	2.4	12	0.79	2.13E-05
8	0.2	2.8	19	0.92	2.37E-07
9	0.2	1.2	12	0.45	0.0116
10	0.2	1.4	18	0.72	1.23 E-04

Table S3: Intraclass correlation coefficient (ICC) and fixed and random-effects linear mixed model variables for inhibition duration at different firing rate groups with varying motoneuron capacitance for the simulated data from cutaneous silent period (CSP) from the first dorsal interosseous (FDI) muscle

	**ICC capacitance**	
**Group**	**Optimized parameters subject 4**	**Optimized parameters subject 8**
11-12Hz	0.16	0.16
12-13Hz	-0.24	-0.23
13-14Hz	0.06	0.02
	**Model:**<br>**Inhibition Duration ~ 1 + Firing rate + (1 | Capacitance)**	
	**Optimized parameters subject 4**	**Optimized parameters subject 8**
**Fixed effects**		
(Intercept)<br>11-12Hz	109 ms [106, 111]<br>$p = 1.47\times10^{-120}$	99 ms [97, 102]<br>$p = 5.14\times10^{-123}$
12-13Hz	-12 ms [-16, -9]<br>$p = 4.00\times10^{-10}$	-8 ms [-11, -5]<br>$p = 1.41\times10^{-6}$
13-14Hz	-18 ms [-22, -15]<br>$p = 3.23\times10^{-19}$	-13 ms [-16, -10]<br>$p = 7.02\times10^{-14}$
**Random effects**		
Capacitance	0 [,] ICC = 0.00	0.98 [0.01, 90] ICC = 0.12
Residuals	9 [8, 10] ICC = 1.00	7.41 [6.46, 8.50] ICC = 0.88
**Model Fit**		
R^2	0.42	0.33

Confidence interval for *Capacitance* random effect could not be accurately estimated.

Table S4: Fixed and random-effects variables and likelihood ratio test from random intercept (model 1) and random intercept and random slope (model 2) linear mixed models for the relationship between Inhibition Duration and firing rate covariation for reciprocal inhibition measured from tibialis anterior (TA) muscle

	**Model 1: Inhibition Duration ~ 1 + Firing rate + (1 | Subject)**	**Model 2: Inhibition Duration ~ 1 + Firing rate + (Firing rate | Subject)**
**Fixed effects**		
(Intercept)	71 ms [61, 81]<br>$p = 3.01\times10^{-26}$	74 ms [64, 83]<br>$p = 8.05\times10^{-31}$
Firing rate	-3 ms [-4, -2]<br>$p = 1.27\times10^{-10}$	-3 ms [-4, -2]<br>$p = 1.15\times10^{-10}$
**Random effects**		
Subject	9 [5, 15], ICC = 0.60	5 [1, 27], ICC = 0.44
Subject x Firing Rate	--	0.3 [0, 4], ICC = 0.03
Correlation (Subject, Firing rate)	--	1 [Singular fit]
Residuals	6 [5, 7], ICC = 0.40	6 [5, 6], ICC = 0.53
**Model Fit and Comparison**		
R^2	0.56	0.56
AIC	800.63	803.83
BIC	811.78	820.55
Log-Likelihood	-396.31	-395.91
p-value	--	0.669 (Model 1 preferred)

AIC: Akaike information criterion; BIC: Bayesian Information Criterion; ICC: Intraclass correlation coefficient

Table S5: Amplitude, time constant and common input values for the LM_{sim} selected from the reciprocal inhibition simulations, with respective R^2 and p value from simulated MUs

Subject	Excitatory input (a.u.)	Inhibitory input amplitude (a.u.)	Inhibitory input time constant (ms)	R^2	p-value
1	0.15	1.2	5	0.33	0.42
2	0.2	1.6	2	0.08	0.71
3	0.15	2.6	4	0.12	0.29
4	0.15	2.4	4	0.02	0.71
5	0.15	1.4	2	0.45	0.10
6	0.15	2	2	0.78	0.31
7	0.2	2.4	3	0.35	0.03
8	0.15	1.8	3	0.18	0.35

Table S6: Parameter values adopted in the *in silico* modelling of S-type motoneurons.

Variable	Definition	Value
l_D	Dendrite length	5.50–6.80mm
r_D	Dendrite radius	20.75–31.25µm
l_S	Soma length	77.50–82.50µm
r_S	Soma radius	38.75–41.25µm
C_m	Membrane specific capacitance	1µF/cm²
g_{Na}	Maximum sodium channel conductance	$30mS/cm^2$
g_{Kf}	Maximum fast potassium channel conductance	$4mS/cm^2$
g_{Kf}	Maximum slow potassium channel conductance	$16\text{-}25mS/cm^2$
g_{Ca}	Maximum calcium channel conductance	$0.038\text{-}0.029\ mS/cm^2$
E_{Na}	Sodium reversal potential channels	120 mV
E_k	Potassium reversal potential channels	-10mV
E_{Ca}	Calcium reversal potential channels	140mV
a_M	Rate constant of sodium channels (closed to open)	$22ms^{-1}$
b_M	Rate constant of sodium channels (open to closed)	$13ms^{-1}$
a_H	Rate constant of sodium channels (non-inactivated to inactivated state)	$0.5ms^{-1}$
b_H	Rate constant of sodium channels (inactivated to non-inactivated state)	$4ms^{-1}$
a_N	Rate constant of sodium channels (closed to open)	$1.5ms^{-1}$
b_N	Rate constant of sodium channels (open to closed)	$0.1ms^{-1}$
a_Q	Rate constant of slow potassium channels (closed to open)	$1.5ms^{-1}$
b_Q	Rate constant of slow potassium channels (open to closed)	$0.025\text{-}0.038ms^{-1}$
a_P	Rate constant of calcium channels (closed to open)	$0.008ms^{-1}$
b_P	Rate constant of calcium channels (open to closed)	$0.014\ \text{-}0.016ms^{-1}$

$C_{dendrite} = 2\pi . r_D . l_D . C_m$

$C_{soma} = 2\pi . r_S . l_S . C_m$

Table S7: Fixed and random-effects variables and likelihood ratio test from random intercept (model 1) and random intercept and random slope (model 2) linear mixed models for the relationship between PSTH inhibition amplitude and firing rate for cutaneous silent period (CSP) measured from first dorsal interosseous (FDI) muscle

	**Model 1:** **PSTH amplitude ~ 1 + Firing rate + (1 | Subject)**	**Model 2:** **PSTH amplitude ~ 1 + Firing rate + (Firing rate | Subject)**
**Fixed effects**		
(Intercept)	-0.242 [-0.452, -0.032]<br>p = 0.024	-0.241 [-0.445, -0.037]<br>p = 0.021
Firing rate	-0.013 [-0.030, 0.003]<br>p = 0.114	-0.013 [-0.030, -10]<br>p =$1.26{\times}10^{-7}$
**Random effects**		
Subject	0.106 [0.061, 0.183], ICC = 0.48	0.081 [0.047, 0.141], ICC = 0.41
Subject x Firing Rate	--	0.002 [0.001, 0.003], ICC = 0.01
Correlation (Subject, Firing rate)	--	1.00 (Singular fit)
Residuals	0.115 [0.096, 0.138], ICC = 0.52	0.115 [0.096, 0.138], ICC = 0.58
**Model Fit and Comparison**		
R^2	0.49	0.49
AIC	-66.02	-62.06
BIC	-57.14	-48.74
Log-Likelihood	37.01	37.03
p-value	--	0.982 (Model1 preferred)

AIC: Akaike information criterion; BIC: Bayesian Information Criterion; ICC: Intraclass correlation coefficient; PSTH – post-stimulus time histogram

Table S8: Fixed and random-effects variables and likelihood ratio test from random intercept (model 1) and random intercept and random slope (model 2) linear mixed models for the relationship between PSF inhibition amplitude and firing rate for cutaneous silent period (CSP) measured from first dorsal interosseous (FDI) muscle

	**Model 1: PSF amplitude ~ 1 + Firing rate + (1 | Subject)**	**Model 2: PSF amplitude ~ 1 + Firing rate + (Firing rate | Subject)**
**Fixed effects**		
(Intercept)	-111 [-203, -21]<br>p = 0.016	-111 [-254, 31]<br>p = 0.122
Firing rate	-4 [-11, 3]<br>p = 0.234	-4 [-15, 6]<br>p =0.384
**Random effects**		
Subject	63 [37, 105], ICC = 0.58	0.178 [65, 492], ICC = 0.76
Subject x Firing Rate	--	12 [4, 38], ICC = 0.05
Correlation (Subject, Firing rate)	--	-0.93 [-0.99, -045]
Residuals	46 [39, 56], ICC = 0.42	43 [35, 53], ICC = 0.18
**Model Fit and Comparison**		
R^2	0.68	0.74
AIC	755.05	758.03
BIC	763.93	771.34
Log-Likelihood	-373.52	-373.01
p-value	--	0.600 (Model1 preferred)

AIC: Akaike information criterion; BIC: Bayesian Information Criterion; ICC: Intraclass correlation coefficient; PSF – peristimulus frequencygram

Table S9: Fixed and random-effects variables and likelihood ratio test from random intercept (model 1) and random intercept and random slope (model 2) linear mixed models for the relationship between PSTH inhibition amplitude and firing rate for reciprocal inhibition measured from tibialis anterior (TA) muscle

	**Model 1:**<br>**PSTH amplitude ~ 1 + Firing rate + (1 | Subject)**	**Model 2:**<br>**PSTH amplitude ~ 1 + Firing rate + (Firing rate | Subject)**
**Fixed effects**		
(Intercept)	-0.163 [-0.207, -0.119]<br>p = 3.32×10^{-11}	-0.148 [-0.198, -0.099]<br>p = 2.02×10^{-8}
Firing rate	0.004 [-0.0003, 0.008]<br>p = 0.075	0.002 [-0.002, 0.005]<br>p = 0.281
**Random effects**		
Subject	0.024 [0.013, 0.045], ICC = 0.45	0.049 [0.038, 0.063], ICC = 0.61
Subject x Firing Rate	--	0.002 [,], ICC = 0.03
Correlation (Subject, Firing rate)	--	-1.00 (Singular fit)
Residuals	0.029 [0.025, 0.033], ICC = 0.55	0.029 [0.025, 0.033], ICC = 0.36
**Model Fit and Comparison**		
R^2	0.35	0.37
AIC	-471.13	-468.61
BIC	-459.98	-451.89
Log-Likelihood	239.56	240.31
p-value	--	0.475 (Model 1 preferred)

AIC: Akaike information criterion; BIC: Bayesian Information Criterion; ICC: Intraclass correlation coefficient; PSTH – post-stimulus time histogram; Confidence interval for Subject x Firing Rate random effect could not be accurately estimated.

Table S10: Fixed and random-effects variables and likelihood ratio test from random intercept (model 1) and random intercept and random slope (model 2) linear mixed models for the relationship between PSF inhibition amplitude and firing rate for reciprocal inhibition measured from tibialis anterior (TA) muscle

	**Model 1:** **PSF amplitude ~ 1 + Firing rate + (1 | Subject)**	**Model 2:** **PSF amplitude ~ 1 + Firing rate + (Firing rate | Subject)**
**Fixed effects**		
(Intercept)	17 [-3 , 39] p = 0.101	-9 [-38 , 21] p = 0.570
Firing rate	-4 [-6, -2] $p = 1.70\times10^{-4}$	-1 [-5, 3] p =0.766
**Random effects**		
Subject	15 [9, 27], ICC = 0.54	30 [11, 84], ICC = 0.64
Subject x Firing Rate	--	5 [2, 10], ICC = 0.11
Correlation (Subject, Firing rate)	--	-1 [Singular fit]
Residuals	13 [11, 15], ICC = 0.46	12 [11, 14], ICC = 0.25
**Model Fit and Comparison**		
R^2	0.73	0.76
AIC	993.98	982.52
BIC	1005.10	999.24
Log-Likelihood	-492.99	-485.26
p-value	--	4.38×10^{-4} (Model 2 preferred)

AIC: Akaike information criterion; BIC: Bayesian Information Criterion; ICC: Intraclass correlation coefficient; PSF – peristimulus frequencygram

Table S11: Fixed and random-effects variables and likelihood ratio test from random intercept (model 1) and random intercept and random slope (model 2) linear mixed models for the simulated data pertaining to the LM_{sim} chosen for each subject, showing the relationship between Inhibition duration and firing rate for simulated cutaneous silent period (CSP)

	**Model 1:**<br>**Inhibition Duration ~ 1 + Firing rate + (1 | Subject)**	**Model 2:**<br>**Inhibition Duration ~ 1 + Firing rate + (Firing rate | Subject)**
**Fixed effects**		
(Intercept)	288 ms [265, 311]<br>$p = 1.97\times10^{-50}$	289 ms [251, 328]<br>$p = 7.65\times10^{-30}$
Firing rate	-14 ms [-16, -12]<br>$p = 8.73\times10^{-31}$	-14 ms [-27, -11]<br>$p = 2.85\times10^{-17}$
**Random effects**		
Subject	17 [10, 28], ICC = 0.57	53 [28, 98], ICC = 0.77
Subject x Firing Rate	--	4 [2, 7], ICC = 0.06
Correlation (Subject, Firing rate)	--	-0.96 [-0.99, -0.80]
Residuals	13 [11, 15], ICC = 0.43	12 [11, 14], ICC = 0.17
**Model Fit and Comparison**		
R^2	0.77	0.80
AIC	1090.9	1086.9
BIC	1102.4	1104.2
Log-Likelihood	-541.45	-537.46
p-value	--	0.019 (Model 2 preferred)

AIC: Akaike information criterion; BIC: Bayesian Information Criterion; ICC: Intraclass correlation coefficient

Table S12: Fixed and random-effects variables and likelihood ratio test from random intercept (model 1) and random intercept and random slope (model 2) linear mixed models for the simulated data pertaining to the LM_{sim} chosen for each subject, showing the relationship between PSTH inhibition amplitude and firing rate for simulated cutaneous silent period (CSP)

	**Model 1:**<br>**PSTH amplitude ~ 1 + Firing rate + (1 | Subject)**	**Model 2:**<br>**PSTH amplitude ~ 1 + Firing rate + (Firing rate | Subject)**
**Fixed effects**		
(Intercept)	-0.532 [-0.655, -0.408]<br>$p = 3.84\times10^{-14}$	-0.538 [-0.685, -0.390]<br>$p = 3.84\times10^{-11}$
Firing rate	0.007 [0.002, 0.012]<br>p = 0.009	0.008 [0.0004, 0.015]<br>p = 0.036
**Random effects**		
Subject	0.168 [0.106, 0.268], ICC = 0.80	0.212 [0.120, 0.373], ICC = 0.95
Subject x Firing Rate	--	0.002 [0.001, 0.003], ICC = 0.01
Correlation (Subject, Firing rate)	--	-0.64 [-0.94, 0.22]
Residuals	0.041 [0.035, 0.045], ICC = 0.20	0.009 [0.002, 0.021], ICC = 0.03
**Model Fit and Comparison**		
R^2	0.94	0.95
AIC	-400.82	-399.26
BIC	-389.32	-382.01
Log-Likelihood	204.41	205.63
p-value	--	0.296 (Model1 preferred)

AIC: Akaike information criterion; BIC: Bayesian Information Criterion; ICC: Intraclass correlation coefficient; PSTH – post-stimulus time histogram

Table S13: Fixed and random-effects variables and likelihood ratio test from random intercept (model 1) and random intercept and random slope (model 2) linear mixed models for the simulated data pertaining to the LM_{sim} chosen for each subject, showing the relationship between PSF inhibition amplitude and firing rate for simulated cutaneous silent period (CSP)

	**Model 1:**<br>**PSF amplitude ~ 1 + Firing rate + (1 | Subject)**	**Model 2:**<br>**PSF amplitude ~ 1 + Firing rate + (Firing rate | Subject)**
**Fixed effects**		
(Intercept)	-98 [-150, -48]<br>p = $1.94{\times}10^{-4}$	-99 [-178, -19]<br>p = 0.015
Firing rate	-6 [-9, -3]<br>p = $4.24{\times}10^{-4}$	-6 [-12, 0.3]<br>p = 0.064
**Random effects**		
Subject	54 [34, 87], ICC = 0.69	115 [65, 204], ICC = 0.79
Subject x Firing Rate	--	9 [5, 16], ICC = 0.06
Correlation (Subject, Firing rate)	--	-0.88 [-0.97, -0.55]
Residuals	24 [21, 27], ICC = 0.31	21 [19, 24], ICC = 0.14
**Model Fit and Comparison**		
R^2	0.84	0.87
AIC	1257.4	1247.7
BIC	1268.9	1265.0
Log-Likelihood	-624.72	-617.87
p-value	--	0.001 (Model 2 preferred)

AIC: Akaike information criterion; BIC: Bayesian Information Criterion; ICC: Intraclass correlation coefficient; PSF – peristimulus frequencygram

Table S14: Fixed and random-effects variables and likelihood ratio test from random intercept (model 1) and random intercept and random slope (model 2) linear mixed models for the simulated data pertaining to the LM_{sim} chosen for each subject, showing the relationship between Inhibition Duration and firing rate for simulated reciprocal inhibition

	**Model 1:**<br>**Inhibition Duration ~ 1 + Firing rate + (1 | Subject)**	**Model 2:**<br>**Inhibition Duration ~ 1 + Firing rate + (Firing rate | Subject)**
**Fixed effects**		
(Intercept)	73 ms [49, 97]<br>$p = 1.26 \times 10^{-7}$	77 ms [52, 101]<br>$p = 3.77 \times 10^{-8}$
Firing rate	-3 ms [-5, -1]<br>p = 0.006	-4 ms [-6, -2]<br>p = 0.003
**Random effects**		
Subject	9 [5, 16], ICC = 0.53	6 [5, 7], ICC = 0.42
Subject x Firing Rate	--	0.3 [0, 2], ICC = 0.02
Correlation (Subject, Firing rate)	--	1 [Singular fit]
Residuals	8 [7, 10], ICC = 0.47	8 [7, 10], ICC = 0.56
**Model Fit and Comparison**		
R^2	0.48	0.48
AIC	441.16	445.08
BIC	449.40	457.44
Log-Likelihood	-216.58	-216.54
p-value	--	0.959 (Model 1 preferred)

AIC: Akaike information criterion; BIC: Bayesian Information Criterion; ICC: Intraclass correlation coefficient

Table S15: Fixed and random-effects variables and likelihood ratio test from random intercept (model 1) and random intercept and random slope (model 2) linear mixed models for the simulated data pertaining to the LM_{sim} chosen for each subject, showing the relationship between PSTH inhibition amplitude and firing rate for simulated reciprocal inhibition

	**Model 1:**<br>**PSTH amplitude ~ 1 + Firing rate + (1 | Subject)**	**Model 2:**<br>**PSTH amplitude ~ 1 + Firing rate + (Firing rate | Subject)**
**Fixed effects**		
(Intercept)	-0.085 [-0.153, -0.017]<br>p = 0.014	-0.096 [-0.174, -0.018]<br>p = 0.015
Firing rate	-0.002 [-0.009, 0.003]<br>p = 0.358	-0.002 [-0.010, 0.006]<br>p = 0.653
**Random effects**		
Subject	0.028 [0.016, 0.051], ICC = 0.56	0.041 [,], ICC = 0.59
Subject x Firing Rate	--	0.007 [0.005, 0.008], ICC = 0.10
Correlation (Subject, Firing rate)	--	-1.00 (Singular fit)
Residuals	0.022 [0.018, 0.027], ICC = 0.44	0.022 [0.018, 0.026], ICC = 0.31
**Model Fit and Comparison**		
R^2	0.60	0.62
AIC	-240.93	-239.01
BIC	-232.69	-226.65
Log-Likelihood	124.47	125.51
p-value	--	0.352 (Model 1 preferred)

AIC: Akaike information criterion; BIC: Bayesian Information Criterion; ICC: Intraclass correlation coefficient; PSTH – post-stimulus time histogram; Confidence interval for Subject in Model 2 could not be accurately estimated.

Table S16: Fixed and random-effects variables and likelihood ratio test from random intercept (model 1) and random intercept and random slope (model 2) linear mixed models for the simulated data pertaining to the LM_{sim} chosen for each subject, showing the relationship between PSF inhibition amplitude and firing rate for simulated reciprocal inhibition

	**Model 1:** **PSF amplitude ~ 1 + Firing rate + (1 | Subject)**	**Model 2:** **PSF amplitude ~ 1 + Firing rate + (Firing rate | Subject)**
**Fixed effects**		
(Intercept)	-4 [-35, 27] p = 0.786	-1 [-32, 29] p = 0.928
Firing rate	-2 [-5, 1] p = 0.254	-2 [-5, 1] p = 0.169
**Random effects**		
Subject	6 [2, 14], ICC = 0.33	11 [8, 15], ICC = 0.47
Subject x Firing Rate	--	0.5 [0.3, 0.7], ICC = 0.02
Correlation (Subject, Firing rate)	--	-1 [Singular fit]
Residuals	12 [10, 14], ICC = 0.67	12 [10, 14], ICC = 0.51
**Model Fit and Comparison**		
R^2	0.18	0.18
AIC	471.94	475.87
BIC	480.18	488.23
Log-Likelihood	-231.97	-231.94
p-value	--	p= 0.966(Model 1 preferred)

AIC: Akaike information criterion; BIC: Bayesian Information Criterion; ICC: Intraclass correlation coefficient; PSF – peristimulus frequencygram

Table S17: Fixed and random-effects variables and likelihood ratio test from random intercept (model 1) and random intercept and random slope (model 2) linear mixed models for the relationship between Firing rate and recruitment threshold for the FDI muscle

	**Model 1:**<br>**Firing rate ~ 1 + Recruitment threshold + (1 | Subject)**	**Model 2:**<br>**Firing rate ~ 1 + Recruitment threshold + (Recruitment threshold | Subject)**
**Fixed effects**		
(Intercept)	13.5 [11.4, 15.5]<br>p = 4.04×10^{-14}	13.4 [11.1, 15.8]<br>p = 9.46×10^{-13}
Firing rate	-3.1 [-5.5, -0.8]<br>p = 0.010	-3.1 [-5.5, -0.6]<br>p = 0.015
**Random effects**		
Subject	1.48 [0.51, 4.28], ICC = 0.38	1.8 [0.6, 6.1], ICC = 0.37
Subject x Recruitment threshold	--	0.7 [0.02, 30.6], ICC = 0.14
Correlation (Subject, Recruitment threshold)	--	-1 [Singular fit]
Residuals	2.43 [1.86, 3.17], ICC = 0.62	2.4 [1.8, 3.1], ICC = 0.49
**Model Fit and Comparison**		
R^2	0.31	0.32
AIC	155.45	159.16
BIC	161.05	167.57
Log-Likelihood	-73.725	-73.58
p-value	--	p= 0.865 (Model 1 preferred)

AIC: Akaike information criterion; BIC: Bayesian Information Criterion; ICC: Intraclass correlation coefficient;

Table S18: Fixed and random-effects variables and likelihood ratio test from random intercept (model 1) and random intercept and random slope (model 2) linear mixed models for the relationship between Firing rate and recruitment threshold for the TA muscle

	**Model 1:** **Firing rate ~ 1 + Recruitment threshold + (1 | Subject)**	**Model 2:** **Firing rate ~ 1 + Recruitment threshold + (Recruitment threshold | Subject)**
**Fixed effects**		
(Intercept)	12.0 [10.9, 12.9]<br>$p = 3.09 \times 10^{-56}$	12.3 [10.7, 13.9]<br>$p = 5.47 \times 10^{-33}$
Firing rate	-2.6 [-3.3, -1.9]<br>$p = 7.13 \times 10^{-12}$	-2.9 [-4.6, -1.2]<br>p = 0.015
**Random effects**		
Subject	1.25 [0.69, 0.2.26], ICC = 0.46	2.14 [1.15, 3.98], ICC = 0.34
Subject x Recruitment threshold	--	2.08 [1.03, 4.18], ICC = 0.33
Correlation (Subject, Recruitment threshold)	--	-0.90 [-0.98, -0.47]
Residuals	1.47 [1.32, 1.62], ICC = 0.54	2.09 [1.03, 4.18], ICC = 0.33
**Model Fit and Comparison**		
R^2	0.49	0.56
AIC	703.69	689.33
BIC	716.59	708.68
Log-Likelihood	-347.84	-338.67
p-value	--	$p = 1.03 \times 10^{-4}$ (Model 2 preferred)

AIC: Akaike information criterion; BIC: Bayesian Information Criterion; ICC: Intraclass correlation coefficient;
